# Substrate-Dependence of Competitive Nucleotide Pyrophosphatase/Phosphodiesterase1 (NPP1) Inhibitors

**DOI:** 10.3389/fphar.2017.00054

**Published:** 2017-02-15

**Authors:** Sang-Yong Lee, Soumya Sarkar, Sanjay Bhattarai, Vigneshwaran Namasivayam, Steven De Jonghe, Holger Stephan, Piet Herdewijn, Ali El-Tayeb, Christa E. Müller

**Affiliations:** ^1^PharmaCenter Bonn, Pharmaceutical Institute, Pharmaceutical Chemistry I, University of BonnBonn, Germany; ^2^Laboratory of Medicinal Chemistry, KU Leuven, Rega Institute for Medical ResearchLeuven, Belgium; ^3^Helmholtz-Zentrum Dresden—Rossendorf, Institute of Radiopharmaceutical Cancer ResearchDresden, Germany

**Keywords:** ectonucleotidase inhibitors, enzyme assay, *p*-nitrophenyl 5′-thymidine monophosphate, NPP1, NPP1 inhibitors, nucleotide pyrophosphatase 1

## Abstract

Nucleotide pyrophosphatase/phosphodiesterase type 1 (NPP1) is a membrane glycoprotein involved in the hydrolysis of extracellular nucleotides. Its major substrate is ATP which is converted to AMP and diphosphate. NPP1 was proposed as a new therapeutic target in brain cancer and immuno-oncology. Several NPP1 inhibitors have been reported to date, most of which were evaluated vs. the artificial substrate *p*-nitrophenyl 5′-thymidine monophosphate (*p*-Nph-5′-TMP). Recently, we observed large discrepancies in inhibitory potencies for a class of competitive NPP1 inhibitors when tested vs. the artificial substrate *p*-Nph-5′-TMP as compared to the natural substrate ATP. Therefore, the goal of the present study was to investigate whether inhibitors of human NPP1 generally display substrate-dependent inhibitory potency. Systematic evaluation of nucleotidic as well as non-nucleotidic NPP1 inhibitors revealed significant differences in determined *K*_*i*_ values for competitive, but not for non- and un-competitive inhibitors when tested vs. the frequently used artificial substrate *p*-Nph-5′-TMP as compared to ATP. Allosteric modulation of NPP1 by *p*-Nph-5′-TMP may explain these discrepancies. Results obtained using the AMP derivative *p*-nitrophenyl 5′-adenosine monophosphate (*p*-Nph-5′-AMP) as an alternative artificial substrate correlated much better with those employing the natural substrate ATP.

## Introduction

**Figure 1:**
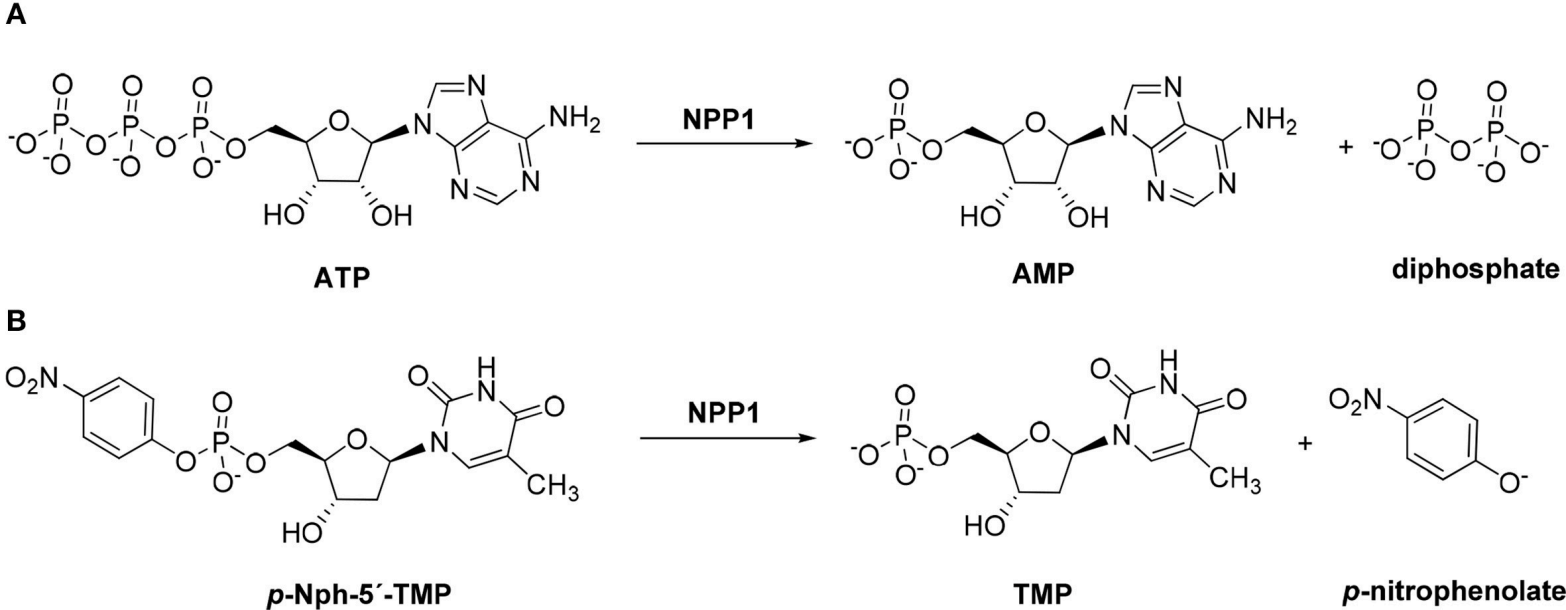
**Hydrolysis of the natural substrate ATP (A) and the artificial substrate *p*-Nph-5′-TMP (B) by NPP1**.

Nucleotide pyrophosphatase/phosphodiesterase 1 (NPP1; PC-1, EC 3.1.4.1) is an enzyme that is attached to the cell membrane, or secreted into the extracellular fluid (Zimmermann et al., 2012). This glycoenzyme is the most important member of the NPP family, which comprises seven closely related proteins, NPP1–7 (Cimpean et al., 2004). Together with ecto-nucleoside triphosphate diphosphohydrolases (NTPDases, EC 3.6.1.5), alkaline phosphatases (APs, EC. 3.1.3.1) and ecto-5′-nucleotidase (eN, CD73, EC. 3.1.3.5) NPPs regulate extracellular levels of nucleotides by catalyzing their hydrolysis eventually leading to the formation of the respective nucleosides and inorganic phosphates (Yegutkin, 2008; Zimmermann et al., 2012). Extracellular nucleosides and nucleotides play an important role as signaling molecules in almost all cell tissues and organs by stimulating P1 (adenosine) and P2 (nucleotide) receptors, respectively. Therefore, ecto-nucleotidases have recently gained considerable interest as novel potential drug targets (Sträter, 2006; Kukulski et al., 2011; Zimmermann et al., 2012; Al-Rashida and Iqbal, 2013). NPP1 cleaves extracellular nucleoside triphosphates releasing nucleoside monophosphates and diphosphate (pyrophosphate, PP_i_); its main substrate is ATP (see Figure 1). In addition, it catalyzes the hydrolysis of dinucleotides (e.g. AP_4_A) and nucleotide sugars, again releasing nucleoside monophosphates (e.g., AMP, UMP) along with the remaining part of the molecule (Zimmermann, 2000; Stefan et al., 2005). cAMP can also be hydrolyzed to AMP by NPP1 (Jackson and Raghvendra, 2004; Sassi et al., 2014; Namasivayam et al., 2017). Recently, the cyclic dinucleotide 2′,3″-cGAMP has been described to be a substrate of NPP1 (Li et al., 2014; Namasivayam et al., 2017).

*p*-Nitrophenyl 5′-thymidine monophosphate (*p*-Nph-5′-TMP, Figure 1) is frequently used as a synthetic substrate for NPP1 to perform kinetic and inhibition assays since the monitoring of enzymatic reactions using natural substrates is much more time-consuming and expensive requiring chromatographic separation or antibodies. The artificial substrate allows colorimetric monitoring of the enzymatic reaction through the formation of the intensively yellow-colored *p*-nitrophenolate, which absorbs at 400 nm (Henz et al., 2007; Buffon et al., 2010). The use of this artificial substrate is popular since it is straightforward and allows high-throughput screening of compound libraries.

NPP1 has been implicated in various physiological and pathological processes. Together with tissue non-specific alkaline phosphatase (TNAP) NPP1 plays an important role in the regulation of bone mineralization and soft-tissue calcification by generating diphosphate (pyrophosphate, PP_i_; Terkeltaub, 2006; Mackenzie et al., 2012). Furthermore, independent of its catalytic activity, NPP1 was reported to downregulate insulin signaling by reducing tyrosine kinase activity of insulin receptors (Abate et al., 2006; Goldfine et al., 2008). NPP1 expression has been reported to be increased in membranes of rat C6 glioma cells (Grobben et al., 2002), human astrocytic brain tumors (Aerts et al., 2011), and human glioblastoma stem-like cells (Bageritz et al., 2014). Increase in NPP1 expression was found to correlate with the aggressiveness of astrocytic brain tumors (Aerts et al., 2011). NPP1 has also been reported to be expressed in N2a mouse neuroblastoma cells, and its expression level was reduced when the cells differentiated into a neuronal-like phenotype (Gómez-Villafuertes et al., 2014). Thus, NPP1 inhibitors might be useful for the treatment of brain cancers. The main substrate of NPP1, ATP, is a proinflammatory signaling molecule. Its concentration is increased in the tumor microenvironment by damaged or dying cells (Antonioli et al., 2013). Along with CD73 NPP1 can convert ATP via AMP to the immunosuppressive signaling molecule adenosine (Horenstein et al., 2013), which is a critical regulator of both innate and adaptive immune responses by stimulating G_s_ protein-coupled A_2A_ and A_2B_ adenosine receptors (Gessi et al., 2007; Bastid et al., 2013). Extracellular adenosine inhibits macrophages and neutrophils, T- and B-cell triggered NF-kB activation, and the production of a series of cytokines such as interleukin-2 (IL-2), interleukin-4 (IL-4), or interferon-gamma (INF-γ) in diverse immune cells (e.g., mast cells, natural killer T cells, dendritic cells, and T lymphocytes; Colgan et al., 2006; Gessi et al., 2007; Stagg and Smyth, 2010; Bergamin et al., 2012; Ghiringhelli et al., 2012; Bastid et al., 2013). Additionally, extracellular adenosine facilitates the differentiation of native T cells into regulatory T cells (T_reg_ cells), which leads to a drastically impaired antitumor immune response (Stagg and Smyth, 2010; Bastid et al., 2013). The inhibition of NPP1 may reduce the formation of extracellular adenosine by diminishing the concentration of extracellular AMP. At the same time it prevents the hydrolysis of ATP, which may directly promote phagocytosis and immunogenicity of the immune cells by stimulating certain P2 receptors (Burnstock and Di Virgilio, 2013; Burnstock and Boeynaems, 2014). Moreover, the blockade of NPP1 may lead to an increase in the concentration of 2′,3″-cGAMP, the agonist of STING, resulting in an increased formation of type 1 interferons (Barber, 2014; Li et al., 2014; Woo et al., 2015). Thus, NPP1 inhibitors hold high potential for the immunotherapy of cancer.

**Figure 2:**
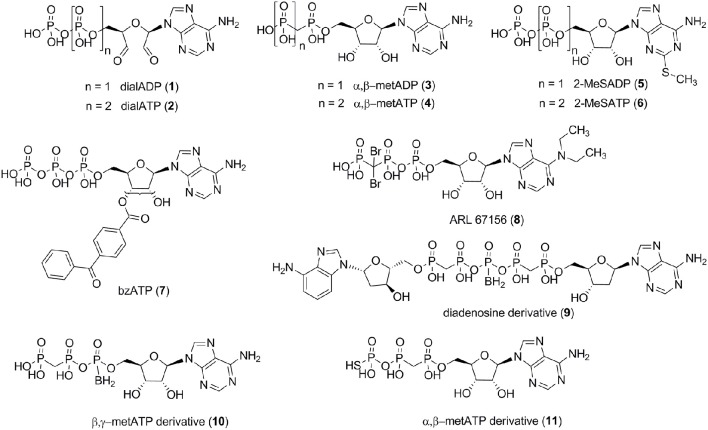
**NPP1 inhibitors with nucleotide structure derived from natural substrates**.

Several small molecule NPP1 inhibitors have been described in the literature. One class of inhibitors is derived from the natural substrates and represents adenine nucleotide derivatives or analogs (see Figure 2). Adenosine 5′-diphosphate-2′,3′-dialdehyde (dialADP, **1**), adenosine 5′-triphosphate-2′,3′-dialdehyde (dialATP, **2**), adenosine 5′-(α,β-methylene)diphosphate (α,β-metADP, **3**), adenosine 5′-(α,β-methylene)triphosphate (α,β-metATP, **4**), 2-methylthio-adenosine 5′-diphosphate (2-MeSADP, **5**), 2-methylthio-adenosine 5′-triphosphate (2-MeSATP, **6**), and 2′ (3′)-O-(4-benzoylbenzoyl)adenosine 5′-triphosphate (bzATP, **7**) were described as inhibitors of human NPP1 with *K*_*i*_-values in the range of 5–27 μM, determined vs. the natural substrate ATP (Lee and Müller, 2014). Furthermore, the standard NTPDase1 inhibitor N^6^,N^6^-diethyl-β,γ-dibromomethylene-ATP (ARL 67156, **8**) was reported to be a weak inhibitor of human NPP1 with a *K*_*i*_-value of 12 μM when tested vs. *p*-Nph-5′-TMP as a substrate (Lévesque et al., 2007). A series of diadenosine 5′,5″-P1,P5-α,β-methylene-δ,ε-methylene-γ-boranopentaphosphates, tested in an assay employing *p*-Nph-5′-TMP as a substrate and recombinantly NPP1-expressing membrane preparations of COS-7 cells as the enzyme source, showed similarly high inhibitory potency at human NPP1, the most potent inhibitor, compound **9** (Figure 2), displaying a *K*_*i*_-value of 9 μM (Eliahu et al., 2010). Subsequently, a new series of metabolically stable ATP analogs, β,γ-methylene-ATP derivatives bearing an α-borano group (e.g., **10**, Figure 2), was developed and optimized as NPP1 inhibitors. They were tested vs. *p*-Nph-5′-TMP as a substrate, and the best compound displayed a *K*_*i*_-value of 0.5 μM (Lecka et al., 2013). Recently, a new series of α,β-methylene-ATP derivatives was investigated, and potent inhibitors for human NPP1, determined vs. *p*-Nph-5′-TMP, were developed, the most potent one being adenosine 5′-γ-thio-α,β-methylene triphosphate (**11**) which showed a *K*_*i*_-value of 0.02 μM (Nadel et al., 2014). The nucleotide derivatives and analogs were shown to display a competitive mechanism of NPP1 inhibition (Laketa et al., 2010; Lecka et al., 2013; Lee and Müller, 2014). Only the dialdehyde derivatives obtained by oxidation of adenine nucleotides, i.e., dialADP (**1**) and dialATP (**2**), were reported to inhibit NPP1 in a non-competitive (**1**) or uncompetitive manner (**2**) (Lee and Müller, 2014).

**Figure 3:**
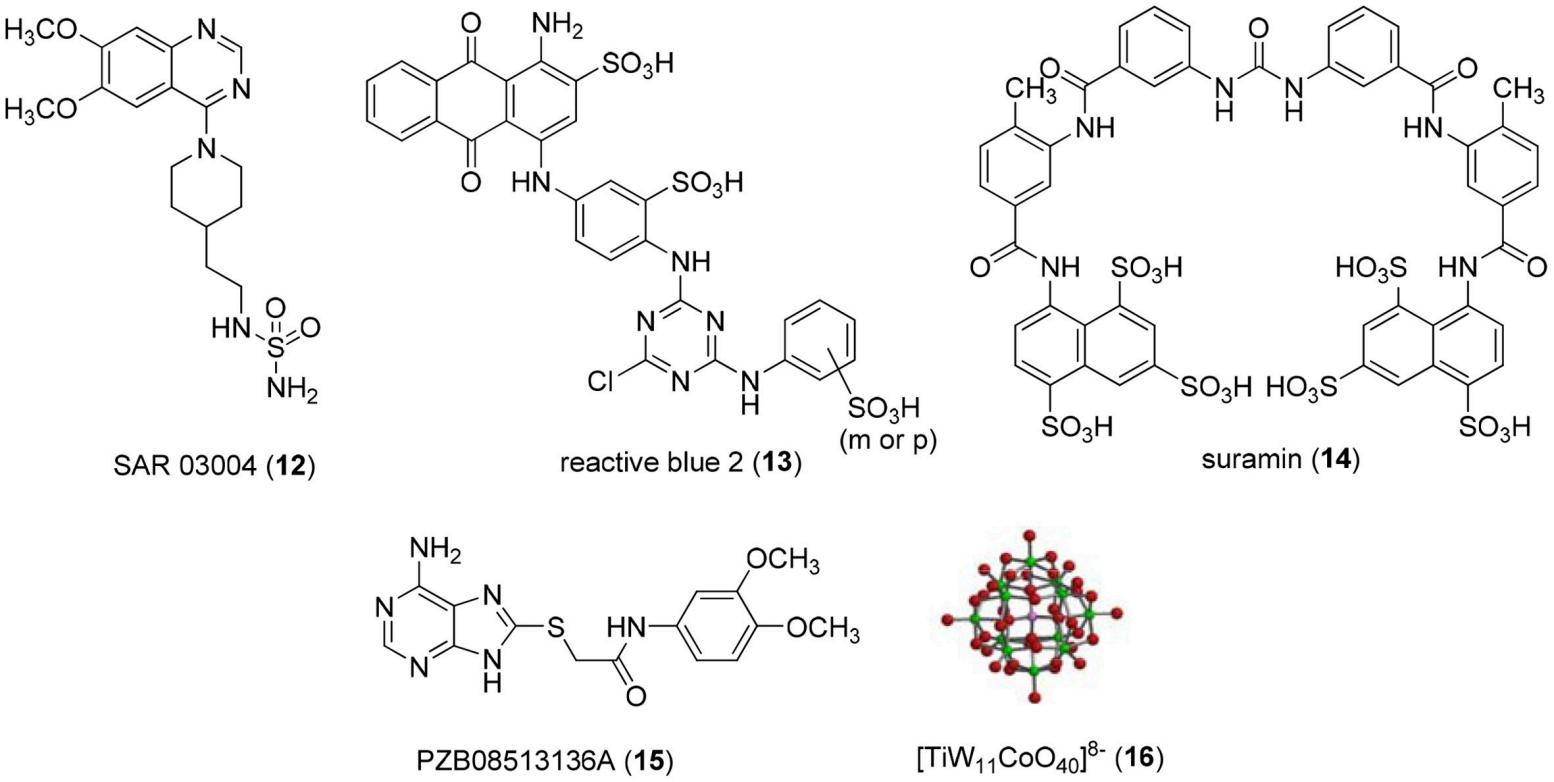
**Potent, non-nucleotide-derived NPP1 inhibitors**.

Non-nucleotide-derived NPP1 inhibitors have also been described (see Figure 3). A series of quinazoline-4-piperidine-4-methylsulfamides was reported as potent NPP1 inhibitors tested against ATP as a substrate, the most potent derivative being SAR 03004 (**12**) with an *IC*_*50*_-value of 0.036 μM (Patel et al., 2009). The mechanism of inhibition was not determined in that study. Very recently, **12** was further investigated as an NPP1 inhibitor using a colorimetric assay with *p*-Nph-5′-TMP as a substrate. The study confirmed that the quinazoline derivative possesses high inhibitory potency with a *K*_*i*_-value of 0.059 μM (Shayhidin et al., 2015). However, that class of compounds also showed high affinity binding to hERG potassium channels, which precluded its further development as a drug, since QT prolongation was to be expected as a side-effect (Patel et al., 2009; Shayhidin et al., 2015). The non-selective purine P2 receptor antagonists reactive blue 2 (**13**) and suramin (**14**) were reported to be relatively potent NPP1 inhibitors vs. ATP as a substrate, displaying *K*_*i*_-values of 0.52 and 0.26 μM, respectively (Iqbal et al., 2008). Their mechanism of inhibition has not been reported. Recently, a series of thioacetamide derivatives was developed as potent competitive NPP1 inhibitors vs. *p*-Nph-5′-TMP as a substrate, the most potent derivative PZB08513136A (**15**) displaying a *K*_*i*_-value of 0.00500 μM (Chang et al., 2014). Moreover, inorganic polyoxometalates, e.g., [TiW_11_CoO_40_]^8−^ (PSB-POM141, **16**), were discovered to be potent and selective non-competitive NPP1 inhibitors, the best compound showing a *K*_*i*_-value of 0.00146 μM vs. ATP as a substrate. This compound represents the most potent inhibitor of human NPP1 described to date (Lee et al., 2015).

The majority of reported NPP1 inhibitors has only been investigated under completely unnatural conditions using spectrophotometric assays with *p*-Nph-5′-TMP as an artificial substrate. However, in several studies substrate-dependent inhibitory potencies of various competitive enzyme inhibitors had been observed (Michaud et al., 1997; Hosoda et al., 1999; Schiemann et al., 2012; Ben Henda et al., 2013; Chang et al., 2014; Lee and Müller, 2014). For example, captopril, a competitive inhibitor of angiotensin-I converting enzyme (ACE), was significantly more potent when the synthetic substrates *N*-[3-(2-furyl)acryloyl]-Phe-Gly-Gly (FAPGG) or *N*-hippuryl-His-Leu hydrate (HHL) were used instead of the natural substrate angiotensin-I (Michaud et al., 1997; Ben Henda et al., 2013). Such a discrepancy in inhibitory potencies depending on the substrate was also observed for NPP2 (autotaxin) (Schiemann et al., 2012), an enzyme that is closely related to NPP1 but prefers phospholipids rather than nucleotides as substrates (Aoki et al., 2008). In our laboratory, we recently found that the inhibitory potencies of several nucleotidic inhibitors of NPP1 were significantly lower when tested vs. ATP as compared to the commonly used artificial substrate *p*-Nph-5′-TMP (Lee and Müller, 2014). The non-nucleotide-derived thioacetamides (e.g., compound **15**, Figure 3) displayed a particularly large discrepancy being much more potent vs. *p*-Nph-5′-TMP as a substrate than vs. ATP (more than 100-fold difference for **15**) (Chang et al., 2014). Thus, the goal of the present study was (i) to fundamentally investigate whether substrate-dependent potency of NPP1 inhibitors was a common phenomenon, and (ii) to find a possible explanation for these observations. To this end, we evaluated a wide range of structurally and mechanistically diverse NPP1 inhibitors vs. both, the artificial substrate *p*-Nph-5′-TMP and the natural substrate ATP, considering both, nucleotidic and non-nucleotidic structures. The results were compared and correlation coefficients were calculated. Moreover, we synthesized and evaluated a new artificial substrate, *p*-nitrophenyl 5′-adenosine monophosphate (*p*-Nph-5′-AMP), which is structurally more similar to ATP than the standard artificial substrate. The results of this study will be highly relevant with respect to *in vivo* studies with NPP1 inhibitors and for translational research aimed at drug development.

## Materials and methods

### Materials

Adenosine 5′-(α,β-methylene)diphosphate (α,β-metADP), adenosine 5′-(α,β-methylene)triphosphate (α,β-metATP), adenosine 5′-diphosphate-2′,3′-dialdehyde (dialADP), adenosine 5′-monophosphate (AMP), adenosine 5′-triphosphate (ATP), adenosine 5′-triphosphate-2′,3′-dialdehyde (dialATP), 2-methylthioadenosine 5′-diphosphate (2-MeSADP), 2-methylthioadenosine 5′-triphosphate (2-MeSATP), *p*-nitrophenol, *p*-nitrophenyl 5′-thymidine monophosphate (*p*-Nph-5′-TMP), reactive blue 2, suramin, and uridine 5′-triphosphate (UTP) were obtained from Sigma (Steinheim, Germany). Calcium chloride, magnesium chloride, sodium hydroxide, and zinc chloride were also from Sigma. 2-(*N*-Cyclohexylamino)ethanesulfonic acid (CHES) and tris-(hydroxymethyl)aminomethane (Tris) were from Applichem (Darmstadt, Germany). Disodium hydrogen phosphate was purchased from Carl Roth (Karlsruhe, Germany). 2-(6-Amino-9*H*-purin-8-ylthio)-*N*-(3,4-dimethoxyphenyl)acetamide (PZB08513136A) was synthesized and provided by the group of Prof. Dr. Piet Herdewijn as previously described (Chang et al., 2014). *N*-[2-[1-(6,7-Dimethoxyquinazolin-4-yl)piperidin-4-yl]ethyl]sulfuric diamide (SAR 03004) was synthesized as described in Supporting Information. [TiW_11_CoO_40_]^8−^ (PSB-POM141) (Müller et al., 2006) was provided by Dr. Holger Stephan (Institute of Radiopharmaceutical Cancer Research, Helmholtz-Zentrum Dresden—Rossendorf). Human recombinant soluble NPP1 (Val191—Leu591) expressed in murine myeloma NS0 cells was purchased from R&D Systems GmbH (Wiesbaden, Germany, purity > 95%, purified by using N-terminal His-tag).

### Synthesis of *p*-nitrophenyl 5′-adenosine monophosphate

**Figure 4:**
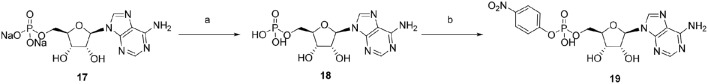
**Synthesis of *p*-nitrophenyl 5 ′-adenosine monophosphate**. Reagents and conditions were as follows: (a) Dowex 50x8 protonated form, 1h; (b) (i) *p*-nitrophenol, dicyclohexylcarbodiimide, triethylamine, pyridine, room temperature, 3 days in the dark; (ii) C-18 RP-HPLC, gradient of acetonitrile: 50 mM aqueous NH_4_HCO_3_ = 0:100–25:75.

*p*-Nitrophenyl 5′-adenosine monophosphate (*p*-Nph-5′-AMP) was synthesized in analogy to a described procedure (Borden and Smith, 1966; Ivanovskaya et al., 1987). Adenosine-5′-monophosphate disodium salt (**17**, see Figure 4, 1.0 g) was dissolved in 20 mL of deionized water. To this solution was added Dowex 50X8 proton form (2 gram) prewashed three times, each with 100 mL of deionized water. The resulting adenosine-5′-monophosphoric acid (**18**, see Figure 4) was separated by filtration and freeze-dried. Adenosine-5′-monophosphoric acid (**18**, 347 mg) was dissolved in pyridine (10 mL) containing triethylamine (1.4 mL). To this was added *p*-nitrophenol (1.4 g) followed by dicyclohexylcarbodiimide (2.0 g), and the mixture was stirred in the dark for 3 days at room temperature, until complete disappearance of adenosine-5′-monophosphosphoric acid as indicated by thin layer chromotagraphy (TLC, solvent system: n-butanol: acetic acid: water = 2:1:1). Pyridine was subsequently removed under reduced pressure, the residue was suspended in water (100 mL), and insoluble dicyclohexylurea was removed by filtration. The aqueous layer was washed three times with diethyl ether (3 × 50 mL) to remove excess *p*-nitrophenol, and the aqueous layer was freeze-dried. Then the freeze-dried crude product was subjected to C-18 reverse phase-HPLC using a gradient of acetonitrile: 50 mM aqueous NH_4_HCO_3_ solution from 0:100 to 25:75 for 50 min. Appropriate fractions were pooled and freeze-dried several times to obtain dry *p*-nitrophenyl 5′-adenosine monophosphate (**19** in Figure 4) as a white solid; yield: 62%. The final product was characterized by LC-MS, ^1^H, ^13^C, and ^31^P NMR spectroscopy. ^1^H NMR (500 MHz, MeOD-*d*_4_): δ 4.23–4.26 (1H, q, H4′, *J* = 5.70 Hz), 4.29–4.36 (2H, m, H5′and H5″CH_2_, *J* = 4.40 Hz), 4.41–4.43 (1H, q, H3′, *J* = 5.05 Hz), 4.70–4.72 (1H, t, H2′, *J*′ = 5.03 Hz and *J*″ = 5.35 Hz), 6.11–6.12 (1H, d, H1′, *J* = 5.35 Hz), 7.38–7.40 (2H, d, Ph-CH_2_, *J* = 9.15 Hz), 8.10–8.12 (2H, d, Ph-CH_2_, *J* = 9.45 Hz), 8.33 (1H, s, H2), 8.56 (1H, s, H8). ^13^C NMR (125 MHz, MeOD-*d*_4_): δ 67.05, 72.30, 76.34, 86.00, 89.85, 120.36, 121.97, 126.45, 143.36, 144.88, 147.92, 150.57, 152.41, 159.91. ^31^P NMR (202 MHz, MeOD-*d*_4_) δ –6.47 (s). LC-ESI-MS: negative mode 467 ([M – H]^−^), positive mode 469 ([M + H]^+^).

### Determination of kinetic parameters of artificial substrates

Enzyme kinetic parameters were measured for *p*-Nph-5′-TMP and *p*-Nph-5′-AMP, both being artificial substrates of human NPP1. Solutions with different concentrations of both substrates (ranging from 1.0 to 500 μM) were prepared in 10 mM CHES buffer (in mM: 1 MgCl_2_, 2 CaCl_2_, 10 CHES, pH 9.0) and added in a final volume of 100 μl to 96-well-plates. The enzyme reactions were initiated by the addition of 20 ng of human NPP1 (for *p*-Nph-5′-TMP), or 75 ng of human NPP1 (for *p*-Nph-5′-AMP). The mixture was incubated at 37°C for 15 min (*p*-Nph-5′-TMP), or 30 min (for *p*-Nph-5′-AMP), respectively, and subsequently terminated by the addition of 20 μl of 1.0 N aqueous NaOH solution. The amounts of *p*-nitrophenolate liberated were measured at 400 nm. Each analysis was repeated twice in three separate experiments.

### Determination of concentration-dependent inhibition curves

Concentration-response curves of NPP1 inhibition were determined for six nucleotidic inhibitors [dialADP (**1**), dialATP (**2**), α,β-metADP (**3**), α,β-metATP (**4**), 2-MeSADP (**5**), and 2-MeSATP (**6**), see Figure 2] and five non-nucleotidic inhibitors [SAR 03004 (**12**), reactive blue 2 (**13**), suramin (**14**), PZB08513136A (**15**), and [TiW_11_CoO_40_]^8−^ (**16**), see Figure 3], and with different substrates. For testing vs. both synthetic substrates, *p*-Nph-5′-TMP and *p*-Nph-5′-AMP, solutions containing different concentrations of each inhibitor were prepared with 10 mM CHES buffer (See Section Determination of Kinetic Parameters of Artificial Substrates) containing 400 μM *p*-Nph-5′-TMP, or *p*-Nph-5′-AMP, respectively, in a final volume of 100 μl. Incubation and operation conditions remained the same as described in Section Determination of Kinetic Parameters of Artificial Substrates, with one exception: reactive blue 2 absorbs light at 400 nm. Therefore, measurements were not performed colorimetrically, but by capillary electrophoresis (CE) with DAD detection of the formed *p*-nitrophenolate at 400 nm. The CE instrumentation and operation conditions were as follows: The CE instrumentation and operating conditions were as follows: P/ACE MDQ capillary electrophoresis system (Beckman Instruments, Fullerton, CA, USA) with a DAD detection system, polyacrylamide-coated capillaries of 50 cm effective length × 50 μm (id) obtained from CS Chromatographie GmbH (Langerwehe, Germany), 100 mM phosphate buffer (pH 6.5) as running buffer, electrokinetic injection (−6 kV, 60 s), separation voltage of −20 kV. The measurement was performed twice in three different experiments. Data collection and peak area analysis were performed by the 32 Karat software obtained from Beckman Coulter (Fullerton, CA, USA). The *IC*_*50*_-values of test compounds for each substrate were calculated by plotting of three independent experiments using the program Prism 5.0 (GraphPad software, San Diego, CA, USA).

For the natural substrate ATP, the enzyme inhibition assays were performed in 10 mM CHES buffer containing 400 μM of substrate along with different inhibitor concentrations. Incubation and operation conditions were the same as described above with artificial substrates. The analysis was performed by CE and the amounts of AMP produced were quantified by their UV absorption at 260 nm. Each analysis was repeated twice in three separate experiments.

### Determination of inhibition constants and mechanism of inhibition

The inhibition mechanisms of the nucleotidic and non-nucleotidic inhibitors were determined using different concentrations of each substrate (from 10 to 1500 μM), and three different concentrations (0, ~0.5- and ~2-fold of *IC*_*50*_-value) of each test compound. The instrumentations and operation conditions for the experiments were the same as those described in the Sections Determination of Kinetic Parameters of Artificial Substrates and Determination of Concentration-Dependent Inhibition Curves. Each analysis was performed in three separate experiments. The inhibition type of each inhibitor was then evaluated graphically from the Hanes-Woolf plots. For the determination of the (α)*K*_*i*_-values the slope of the reciprocal lines from the Hanes-Woolf plot were plotted as a function of inhibitor concentrations using Prism 5.0.

### Molecular docking of artificial substrates

The generated homology model of human NPP1 described in Namasivayam et al. (2017). was used for the docking procedure using AutoDock 4.2 (Morris et al., 2009). The AutoDockTools package was employed to generate the docking input files and to analyze the docking results (Sanner, 1999). The search algorithm Lamarkian genetic algorithm (LGA) and the default scoring function, a hybrid scoring function (semi-empirical and free-energy) was employed for docking calculations. Three-dimensional energy scoring grids for a box of 60 × 60 × 60 points with a spacing of 0.375 Å were computed. The grids were centered based on the co-crystallized ligand, which was transformed into the homology model. A total of 50 runs with a maximum of 250,000 energy evaluations were performed with the default parameters for the genetic algorithm (GA) and Solis-Wet local search, a method that facilitates random moving around the binding pose identified through the GA. High scoring binding poses (of lowest energy) or more populated poses were selected for the analysis on the basis of visual inspection.

### Statistical analyses

Statistical data analyses of *pK*_*i*_-values were performed using Prism 5.0 software. The *pK*_*i*_-values [–log_10_(α)*K*_*i*_-values] were calculated from the obtained (α)*K*_*i*_-values in each assay. Data were tested for statistical significance by one-way ANOVA as appropriate. When significant differences were observed, Tukey multiple comparison tests were performed. A value of *p* < 0.05 was considered significant.

### Calculation of correlation coefficients between assays with different substrates

The correlation coefficients (*R*^2^) were evaluated by comparing *pK*_*i*_-values of one assay to those of another assay using Prism 5.0.

## Results and discussion

The main natural substrate of NPP1, ATP (Figure 1A), and the generally used artificial NPP1 substrate, *p*-Nph-5′-TMP (Figure 1B; Laketa et al., 2010; Lee et al., 2012), differ not only in the phosphoric ester part (triphosphate vs. *p*-nitrophenyl phosphate), but also with respect to their nucleoside partial structure (adenosine vs. thymidine). In order to investigate potential substrate-dependence of various NPP1 inhibitors, we intended to test selected antagonists vs. both substrates. But, in addition, we decided to additionally evaluate them on a second artificial substrate, which is structurally more closely related to ATP, namely *p*-nitrophenyl 5′-adenosine monophosphate (*p*-Nph-5′-AMP, **19**). Since **19** was not commercially available we decided to synthesize the compound.

### Synthesis of a new artificial substrate of NPP1

*p*-Nph-5′-AMP had been previously synthesized (Borden and Smith, 1966; Ivanovskaya et al., 1987), but no detailed characterization of the compound has been published. Initially we tried to prepare **19** directly from adenosine by reaction with *p*-nitrophenyl phosphorodichloridate. However, the reagent is toxic and difficult to handle. Moreover, tedious separation and purification procedures were required to obtain the desired product in sufficient purity, and the yield was only about 20%. Therefore, we decided to utilize commercially available AMP (**17**) for the preparation of **19**. In a one-step reaction AMP was condensed with *p*-nitrophenol in the presence of *N,N*′-dicyclohexylcarbodiimide yielding product **19** in 62% yield after purification (see Figure 4).

### Biochemical characterization and molecular docking of the new artificial substrate

In subsequent experiments, we characterized the newly synthesized alternative substrate *p*-Nph-5′-AMP in comparison with the common artificial substrate *p*-Nph-5′-TMP and the natural substrate ATP. Enzyme kinetic analysis showed the following rank order of substrate preference: ATP > > *p*-Nph-5′-TMP > *p*-Nph-5′-AMP. The *k*_*cat*_/*K*_*m*_-values are 674 × 10^3^ M^−1^s^−1^ (*k*_*cat*_ = 5.51 s^−1^, *K*_*m*_ = 8.17 μM) for ATP (Namasivayam et al., 2017), 100 × 10^3^ M^−1^s^−1^ (*k*_*cat*_ = 22.3 s^−1^, *K*_*m*_ = 222 μM) for *p*-Nph-5′-TMP (Namasivayam et al., 2017), and 13.4 × 10^3^ M^−1^s^−1^ (*k*_*cat*_ = 2.51 s^−1^, *K*_*m*_ = 188 μM) for *p*-Nph-5′-AMP (see Figure S1 in Supporting Information). While the *K*_*m*_-value of *p*-Nph-5′-AMP was almost identical to that of *p*-Nph-5′-TMP indicating similar affinities, the *k*_*cat*_-value determined for *p*-Nph-5′-TMP was nearly 10-fold higher than that for *p*-Nph-5′-AMP. This means that *p*-Nph-5′-TMP is hydrolyzed by NPP1 much faster than *p*-Nph-5′-AMP. Compared to the natural substrate ATP, the new artificial substrate has a similar *k*_*cat*_-value, but its *K*_*m*_-value is > 20-fold higher than that for ATP. This indicates that the binding affinity of *p*-Nph-5′-AMP is significantly lower than that of ATP.

**Figure 5:**
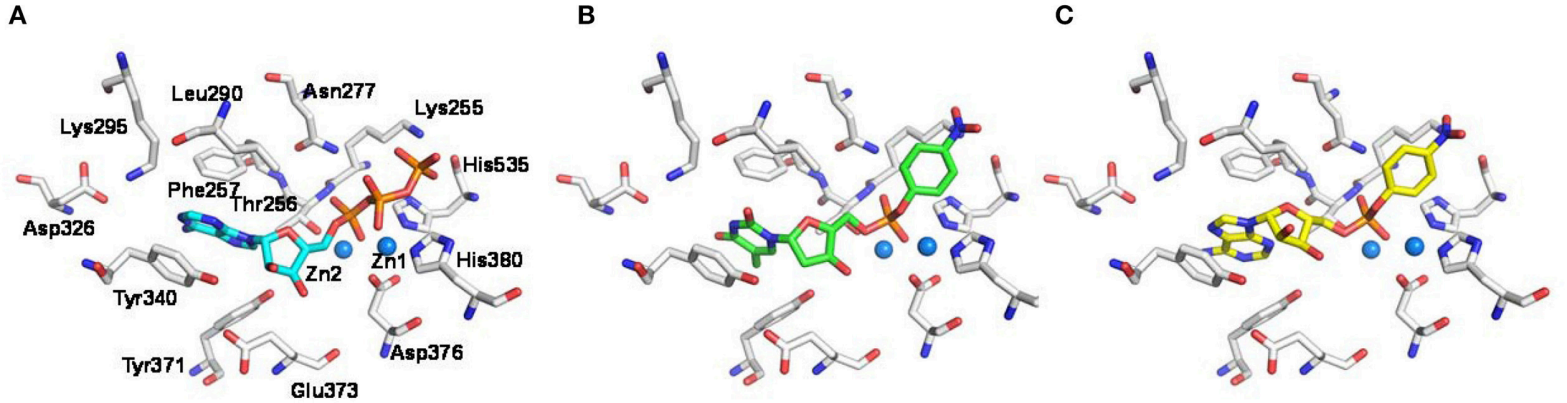
**Putative binding modes of NPP1 substrates**. Docking poses of **(A)** ATP (cyan colored), **(B)** the commonly used artificial substrate *p*-Nph-5′-TMP (green), and **(C)** the new artificial substrate *p*-Nph-5′-AMP (yellow) represented as sticks. Important residues in the binding pocket are shown (white); oxygen atoms are colored in red, nitrogen atoms in blue, and phosphorus atoms in orange. The zinc ions in the active site are represented as spheres (marine blue).

In order to gain insights into the molecular determinants involved in the formation of the enzyme-substrate complex, the new artificial substrate *p*-Nph-5′-AMP was docked into a homology model of the human NPP1 (Namasivayam et al., 2017), which was generated based on the recently solved crystal structure of the mouse NPP1 (Kato et al., 2012). The observed interactions were compared to those of ATP and *p*-Nph-5′-TMP. As shown in Figure 5A, the α-phosphate group of the substrate ATP is bound between the two zinc ions, and the two other phosphate groups form hydrogen bond interactions with the following amino acid residues: Lys255, Thr256, Asn277, His380, and His535. Tyr340 forms a hydrogen bond with the ribose moiety, and the adenine ring of ATP is stacked between Phe257 and Tyr340 (Namasivayam et al., 2017). Similarly, both artificial substrates form complexes with the zinc ions of the enzyme with their phosphate groups as shown in Figures 5B,C. Because the *p*-nitrophenylphosphate group of both artificial substrates interacts in the same way with the zinc ions in the active site of the enzyme, the ground state of binding interactions may be comparable, which explains the similar *K*_*m*_-values determined for both artificial substrates. While *p*-Nph-5′-AMP binding is stabilized through π-π interactions of the adenine base mainly with Tyr340 of the enzyme, this interaction is expected to be weaker for the artificial substrate *p*-Nph-5′-TMP due to the exchange of adenine for thymine. Furthermore, a 2′-hydroxyl group at the ribose moiety is lacking in *p*-Nph-5′-TMP, but not in *p*-Nph-5′-AMP, and therefore, the interaction between that OH group and the side chain of Tyr340 is missing in *p*-Nph-5′-TMP. Overall, *p*-Nph-5′-TMP has less interactions than *p*-Nph-5′-AMP and therefore, it may be hydrolyzed faster via the transition state than the new artificial substrate. This may explain why *p*-Nph-5′-TMP showed a significantly higher *k*_*cat*_-value than *p*-Nph-5′-AMP.

### Characterization of NPP1 inhibitors vs. different substrates

**Figure 6:**
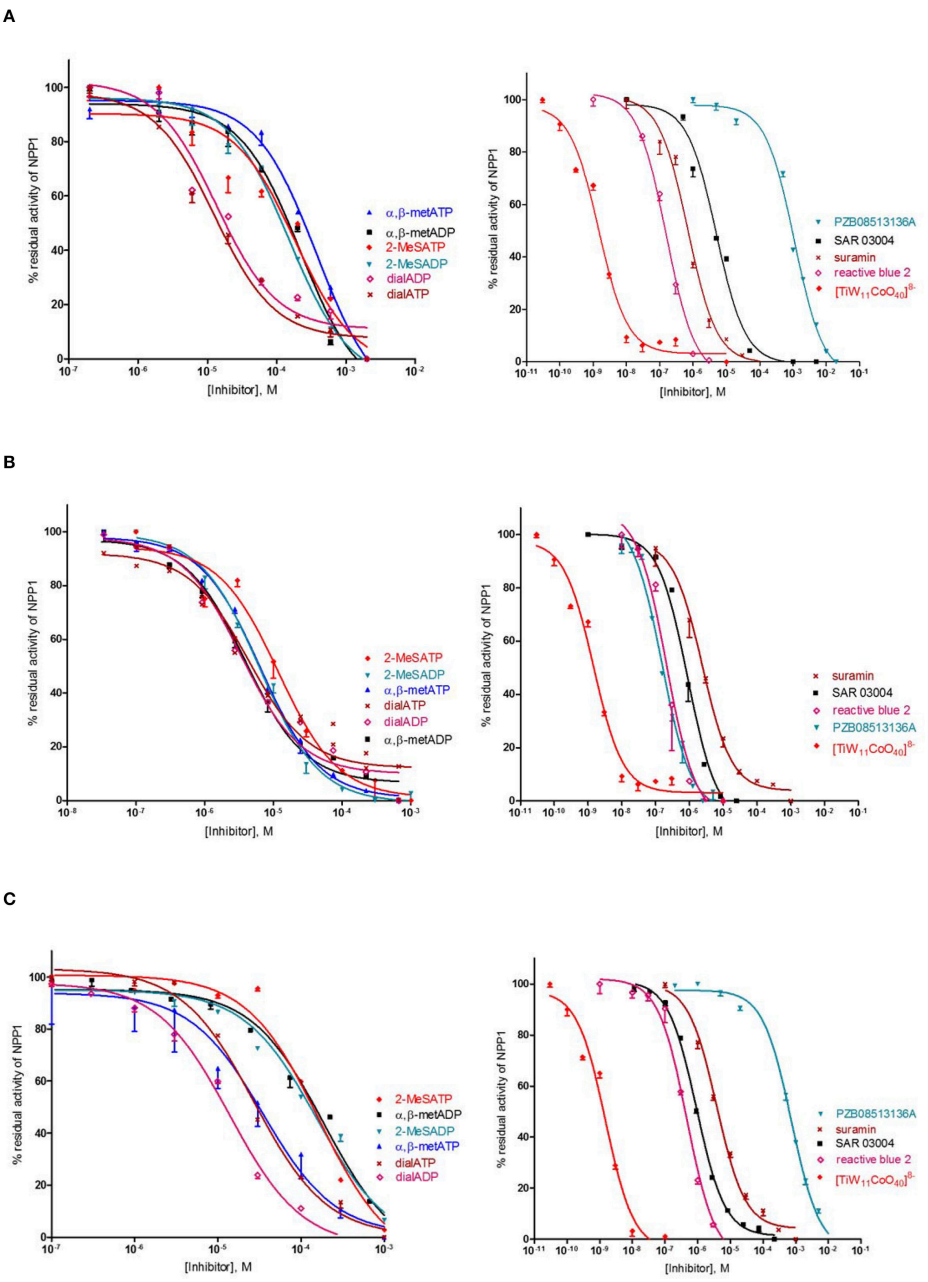
**Concentration-dependent inhibition curves for non-nucleotidic and nucleotidic inhibitors vs. different substrates (A: ATP, B: *p*-Nph-5′-TMP, C: *p*-Nph-5′-AMP)**. Figures represent means ± SD from three independent experiments.

Several previous studies had indicated that the potency of enzyme inhibitors may be dependent on the substrate used for testing (Michaud et al., 1997; Hosoda et al., 1999; Schiemann et al., 2012; Ben Henda et al., 2013; Chang et al., 2014; Lee and Müller, 2014). Therefore, we decided to investigate a selection of standard NPP1 inhibitors vs. three different substrates: six nucleotidic inhibitors, dialADP (**1**), dialATP (**2**), α,β-metADP (**3**), α,β-metATP (**4**), 2-MeSADP (**5**) and 2-MeSATP (**6**), and five non-nucleotidic inhibitors, SAR 03004 (**12**), reactive blue 2 (**13**), suramin (**14**), PZB08513136A (**15**), and [TiW_11_CoO_40_]^8−^ (**16**) (for structures see Figures 2, 3). The concentration-inhibition curves of the test compounds vs. the natural substrate ATP and the two artificial substrates *p*-Nph-5′-TMP and *p*-Nph-5′-AMP are presented in Figure 6. Table 1 displays the (α)*K*_*i*_-values and the determined inhibition types of the NPP1 inhibitors. Six of the investigated inhibitors displayed a competitive mechanism of NPP1 inhibition (also see Figure S2 in Supporting Information). As expected, this was independent of the structure of the substrate and could be confirmed for all three investigated substrates. [TiW_11_CoO_40_]^8−^, reactive blue 2 and dialADP inhibited the enzymatic activity in a non-competitive manner, while suramin and dialATP were characterized as un-competitive inhibitors vs. all investigated substrates. Differences in inhibitory potential with respect to structure of inhibitor also exist. Among the investigated competitive inhibitors, the quinazoline sulfonamide derivative SAR 03004 (**12**) was found to be the most potent compound vs. ATP and also vs. *p*-Nph-5′-AMP with *K*_*i*_-values of 0.215 and 0.420 μM, respectively. To the contrary, when *p*-Nph-5′-TMP was employed as a substrate, the thioacetamide derivative PZB08513136A (**15**) was found to be the most potent competitive inhibitor with a *K*_*i*_-value of 0.00500 μM. Among the investigated non-competitive inhibitors, [TiW_11_CoO_40_]^8−^ (**16**) was the most potent compound vs. all investigated substrates (*K*_*i*_-values: 0.00146 μM vs. ATP, 0.00199 μM vs. *p*-Nph-5′-TMP and 0.00174 μM vs. *p*-Nph-5′-AMP). In the group of un-competitive inhibitors, suramin (**14**) was found to be the most potent compound vs. all investigated substrates (*K*_*i*_-values: 0.780 μM vs. ATP, 1.07 μM vs. *p*-Nph-5′-TMP and 1.03 μM vs. *p*-Nph-5′-AMP). Among the adenosine analogs and derivatives, the two dialdehyde compounds (dialADP; non-competitive inhibitor, dialATP; un-competitive inhibitor) displayed NPP1-inhibitory activities with *K*_*i*_-values around 5 μM vs. all substrates. The other investigated adenine nucleotide derivatives and analogs were mostly less active vs. the three different substrates (*K*_*i*_-values: 13.0–32.8 μM vs. ATP, 1.28–4.47 μM vs. *p*-Nph-5′-TMP and 8.19–39.9 μM vs. *p*-Nph-5′-AMP).

**Table 1:** **(α)*K*_*i*_-values and inhibition types of nucleotidic and non-nucleotidic inhibitors of human NPP1 using different substrates**.

**Inhibitors**	***K_*i*_* ± SD (μM)^a^**			**Inhibition type**
	**ATP**	***p*-Nph-5′-TMP**	***p*-Nph-5′-AMP**	
**NUCLEOTIDIC INHIBITORS**				
dialADP (**1**)	**5.62** ± 1.23	**5.03** ± 0.15	**5.09** ± 1.72	Non-competitive
dialATP(**2**)	**6.82** ± 1.01^b^	**4.09** ± 0.41^b^	**5.08** ± 0.33^b^	Un-competitive
α,β-metADP (**3**)	**16.5** ± 3.1	**1.28** ± 0.16	**25.8** ± 4.1	Competitive
α,β-metATP (**4**)	**13.0** ± 3.0	**3.32** ± 0.51	**8.19** ± 1.32	Competitive
2-MeSADP (**5**)	**32.8** ± 7.0	**2.18** ± 0.29	**35.4** ± 6.4	Competitive
2-MeSATP (**6**)	**25.3** ± 5.9	**4.47** ± 0.66	**39.9** ± 6.9	Competitive
**NON-NUCLEOTIDIC INHIBITORS**				
SAR 03004 (**12**)	**0.215** ± 0.099	**0.0642** ± 0.0192	**0.420** ± 0.090	Competitive
Reactive blue 2 (**13**)	**0.141** ± 0.031	**0.198** ± 0.034	**0.176** ± 0.025	Non-competitive
suramin (**14**)	**0.780** ± 0.081^b^	**1.07** ± 0.23^b^	**1.03** ± 0.22^b^	Un-competitive
PZB08513136A (**15**)	**18.0** ± 2.7^c^	**0.00500** ± 0.00077^c^	**14.9** ± 0.8	Competitive
[TiW_11_CoO_40_]^8−^ (**16**)	**0.00146** ± 0.00001^d^	**0.00199** ± 0.00033	**0.00174** ± 0.00099	Non-competitive

a *Results are expressed as means (in bold) ± SD of three independent experiments*.

b *αK_i_-value*.

c *K_i_-values from the literature (Chang et al., 2014), expressed as means ± SEM*.

d *K_i_-values from the literature (Lee et al., 2015), expressed as means ± SEM*.

### Correlation

**Figure 7:**
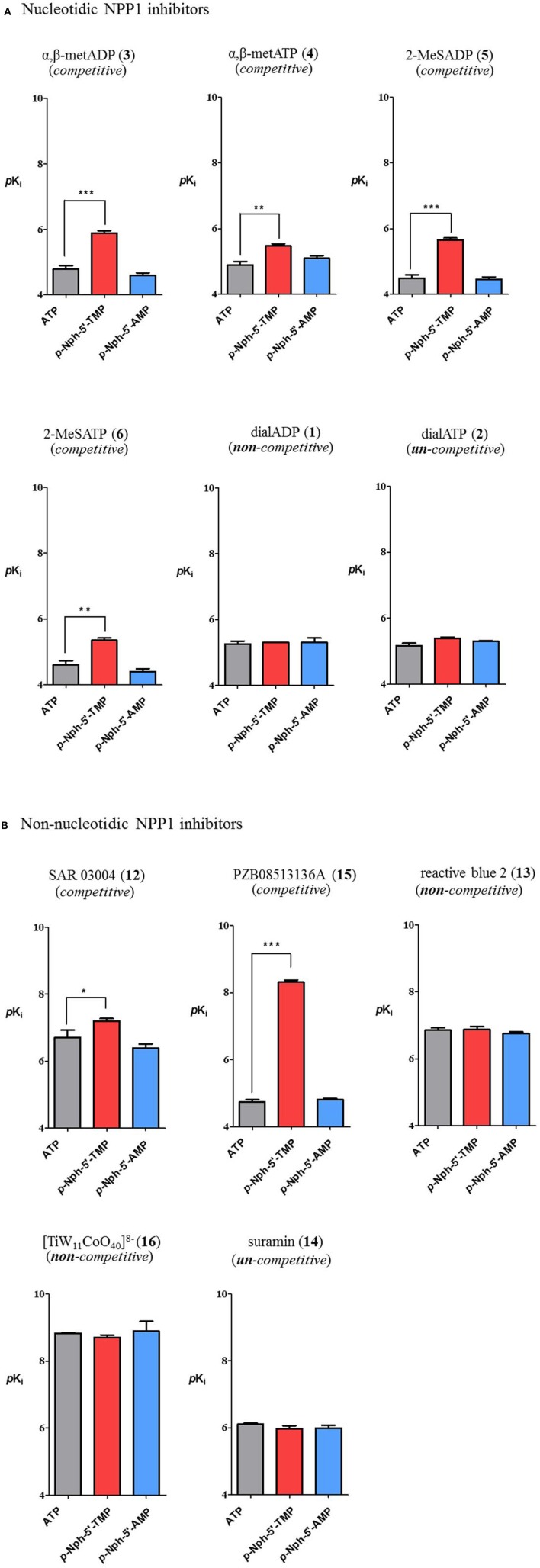
**Comparison of inhibitory potency of nucleotidic (A) and non-nucleotidic (B) inhibitors using different substrates**. Data are means ± *SDs* of *pK*_*i*_-values. The bars in gray represent *pK*_*i*_*-v*alues of inhibitors vs. the natural substrate ATP, those in red are the *pK*_*i*_*-v*alues of inhibitors vs. *p*-Nph-5′-TMP and those in blue are *pK*_*i*_ values vs. *p*-Nph-5′-AMP. ^*^p < 0.05, ^**^p < 0.01, and ^***^p < 0.001.

Inhibitory potencies of non-nucleotidic and nucleotidic inhibitors vs. different substrates were compared (Figure 7). Data analysis revealed substrate-dependent inhibitory potencies of competitive inhibitors, but not of non- or uncompetitive inhibitors. When *K*_*i*_-values obtained with the natural substrate ATP were compared with those obtained with the artificial substrate *p*-Nph-5′-TMP the competitive inhibitors were 3–3600-fold more potent against *p*-Nph-5′-TMP than vs. ATP [*p* < 0.05 for SAR 03004 (**12**), *p* < 0.01 for α,β-metATP (**4**) and 2-MeSATP (**6**), and *p* < 0.001 for α,β-metADP (**3**), 2-MeSADP (**5**) and PZB08513136A (**15**)]. Differences were also dependent on the structure of the competitive antagonists, e.g., it was particularly high for the thioacetamide derivative PZB08513136A (**15**), but less pronounced for the quinazoline derivative SAR 03004 (**12**). In contrast, results obtained vs. the new artificial substrate *p*-Nph-5′-AMP were similar to those obtained vs. the natural substrate ATP. As opposed to competitive inhibitors, substrate-dependent inhibition was not observed for non-competitive and un-competitive inhibitors, and very similar inhibition constants were obtained vs. all investigated substrates.

**Figure 8:**
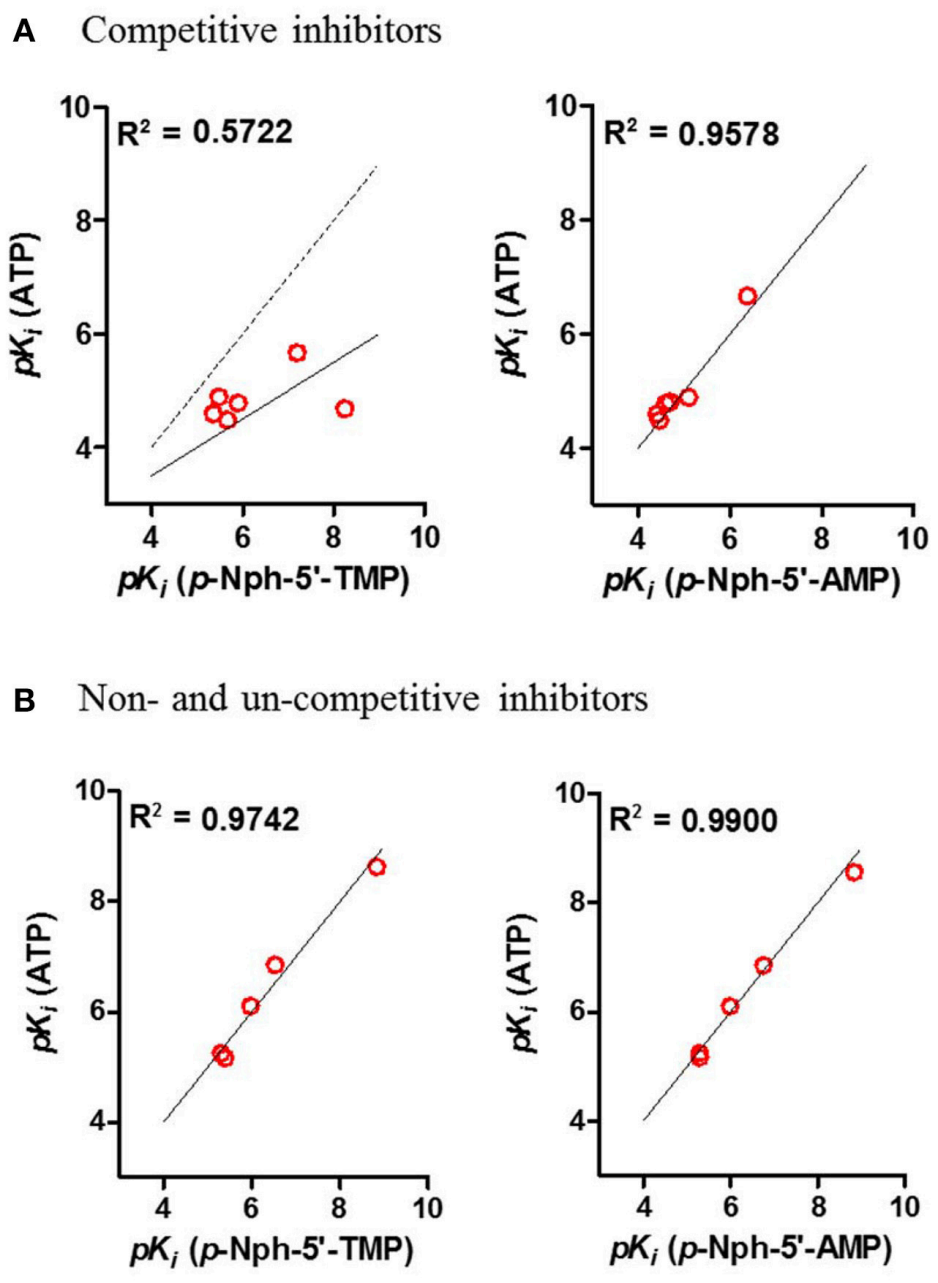
**Correlation analyses between the results (A) for competitive inhibitors, and for (B) non- and un-competitive inhibitors obtained with different substrates**. Determined correlation coefficients (*R*^2^) were calculated by fitting *pK*_*i*_-values obtained with one substrate vs. those obtained with another substrate using the software Prism 5.0; red points, test compounds; solid line, the best fit line of the linear regression; the dotted line in **(A)** represents the ideal correlation (*R*^2^ = 1.00).

Correlation analyses of *pK*_*i*_-values obtained vs. one substrate with those measured vs. another substrate were performed. Considering the competitive inhibitors, a low correlation of data obtained with *p*-Nph-5′-TMP as a substrate with those obtained with the natural substrate ATP was obtained [correlation coefficient (*R*^2^) = 0.5722, see Figure 8A], whereas a high correlation between the results obtained with *p*-Nph-5′-AMP as a substrate and those determined with ATP was observed (*R*^2^ = 0.9578). Moreover, Figure 8A (left) showed that the data points were shifted to the right of the ideal correlation line [dotted line in Figure 8A (left)]. This indicates that the competitive NPP1 inhibitors were generally more potent vs. *p*-Nph-5′-TMP than vs. ATP as a substrate. Contrary to this, the non- and un-competitive inhibitors showed high correlations, no matter which substrates were used for comparison (*R*^2^ = 0.9742 for competitive inhibitors; *R*^2^ = 0.9900 for non- and un-competitive inhibitors), see Figure 8B.

### Possible explanation for substrate-dependence of competitive NPP1 inhibitors

**Figure 9:**
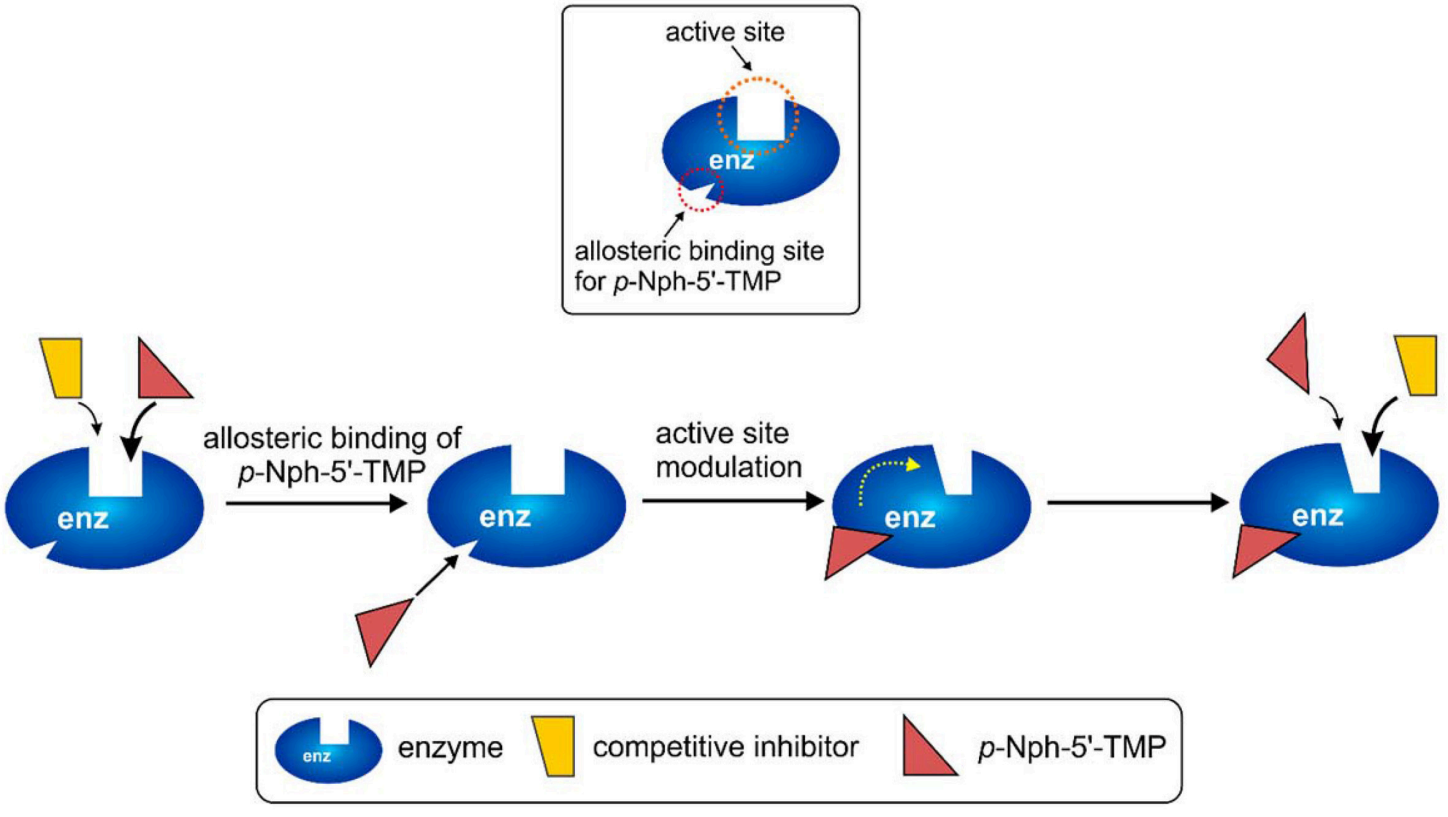
**Possible explanation for the discrepancies observed for competitive inhibitors vs. the artificial substrate *p*-Nph-5′-TMP (higher affinity observed for competitive antagonists) as compared to natural substrates assays (lower affinity for competitive inhibitors)**. *p*-Nph-5′-TMP may not only act as a substrate, but also as an allosteric modulator.

The observation of significantly different potencies of competitive enzyme inhibitors when determined vs. different substrates is puzzling, and an explanation for this phenomenon is not straightforward. The different assay conditions are clearly not the reason for the observed discrepancies because the same operating conditions (e.g., same stock solutions of inhibitors, same assay buffer) were applied for the enzyme inhibition assays with different substrates. A rational explanation for the different results between assays obtained with *p*-Nph-5′-TMP and the natural substrate ATP could be an allosteric modulatory effect by *p*-Nph-5′-TMP on the enzyme, in addition to acting as a substrate (Figure 9). Such allosteric binding of the substrate has previously been reported for another nucleotide-metabolizing enzyme, bacterial UDP-*N*-acetylglucosamine 2-epimerase, which is allosterically modulated by its substrate UDP-*N*-acetylglucosamine (Velloso et al., 2008). The binding of *p*-Nph-5′-TMP to its allosteric binding site, which may be close or even distant from the active site, could induce a conformational change of the substrate binding site. This would modulate the interaction of competitive inhibitors with the substrate binding site, and could therefore explain the increased affinity of the investigated competitive inhibitors (Figure 9). This hypothesis is supported by the fact that the affinity increase depends on the structure of the inhibitors, e.g. some competitive inhibitors (e.g., **15**) being much more strongly affected than others (see also Table 1 and Figure 9).

This hypothesis also provides a straightforward explanation for the finding that *p*-Nph-5′-TMP is a much better NPP1 substrate than *p*-Nph-5′-AMP despite the fact that—based on docking studies—the AMP derivative should have stronger interactions with the substrate binding site. *p*-Nph-5′-TMP may additionally bind to an allosteric site and thereby act as a positive allosteric modulator which increases its binding affinity to the substrate binding site and accelerates its hydrolysis.

Further, investigations to corroborate this hypothesis of allosteric modulation of the active site by *p*-Nph-5′-TMP are warranted.

## Conclusions

In conclusion, we observed substrate-dependence of the inhibitory potency of NPP1 inhibitors, competitive inhibitors being (much) more potent vs. the artificial substrate *p*-Nph-5′-TMP than vs. the natural nucleotide substrate ATP. In contrast, data obtained using the new artificial substrate *p*-Nph-5′-AMP correlated well with those determined vs. the natural substrate ATP indicating that the nucleoside part of the artificial substrate was responsible for the observed effects. No significant differences in inhibitory potencies were observed for non- or un-competitive inhibitors. The most likely explanation for the observed phenomenon is an allosteric modulation of NPP1 by the artificial substrate *p*-Nph-5′-TMP, but not by *p*-Nph-5′-AMP. Therefore, we recommend to use *p*-Nph-5′-AMP instead of *p*-Nph-5′-TMP for high-throughput screening of NPP1 using colorimetric detection. Further, investigations to explain the discrepancy between results with the commonly used artificial substrate *p*-Nph-5′-TMP and the natural substrate ATP are in progress.

## Author contributions

SL performed the pharmacological experiments, analyzed the data and contributed to writing of the manuscript. SS, SB, SD, HS, PH, and AE synthesized, purified and analyzed compounds. VN performed the molecular modeling studies and contributed to writing of the manuscript. CM designed and supervised the study and wrote the manuscript.

### Conflict of interest statement

The authors declare that the research was conducted in the absence of any commercial or financial relationships that could be construed as a potential conflict of interest.

## Abbreviations

α,β-metADP: adenosine 5′-(α,β-methylene)diphosphate
α,β-metATP: adenosine 5′-(α,β-methylene)triphosphate
CE: capillary electrophoresis
CHES: 2-(*N*-cyclohexylamino)-ethanesulfonic acid
dialADP: adenosine 5′-diphosphate-2′,3′-dialdehyde
dialATP: adenosine 5′-triphosphate-2′,3′-dialdehyde
2-MeSADP: 2-methylthioadenosine 5′-diphosphate
2-MeSATP: 2-methylthioadenosine 5′-triphosphate
*p*-Nph-5′-AMP: *p*-nitrophenyl 5′-adenosine monophosphate
*p*-Nph-5′-TMP: *p*-nitrophenyl 5′-thymidine monophosphate
NPP: nucleotide pyrophosphatase/phosphodiesterase
PZB08513136A: 2-(6-amino-9*H*-purin-8-ylthio)-*N*-(3,4-dimethoxyphenyl)acetamide
SAR 03004: *N*-[2-[1-(6,7-dimethoxyquinazolin-4-yl)piperidin-4-yl]ethyl]sulfuric diamide.
