# Supplementary material for: Substrate-Dependence of Competitive Nucleotide Pyrophosphatase/Phosphodiesterase1 (NPP1) Inhibitors
Source: Front Pharmacol. 2017 Feb 15;8:54. doi: 10.3389/fphar.2017.00054 (PMC5309242; doi:10.3389/fphar.2017.00054)
Supplement: Supplementary file 1 [file DataSheet1.PDF]

Supporting Information

Substrate-dependence of competitive nucleotide pyrophosphatase / phosphodiesterase 1 (NPP1) inhibitors

Sang-Yong Lee,[a] Soumya Sarkar,[a] Sanjay Bhattarai,[a] Vigneshwaran Namasivayam,[a] Steven De Jonghe,[b] Holger Stephan,[c] Piet Herdewijn,[b] Ali El-Tayeb,[a] and Christa E. Müller[a,]*

[a] *PharmaCenter Bonn, Pharmaceutical Institute, Pharmaceutical Chemistry I, University of Bonn, An der Immenburg 4, D-53121 Bonn, Germany*

[b] *KU Leuven, Rega Institute for Medical Research, Laboratory of Medicinal Chemistry, Minderbroedersstraat 10, 3000 Leuven, Belgium*

[c] *Institute of Radiopharmaceutical Cancer Research, Helmholtz-Zentrum Dresden - Rossendorf, Bautzner Landstrasse 400, 01328 Dresden, Germany*

* Correspondence**:** Prof. Dr. Christa Müller, PharmaCenter Bonn, Pharmaceutical Sciences Bonn (PSB), Pharmaceutical Institute, Pharmaceutical Chemistry I, University of Bonn, An der Immenburg 4, D-53121 Bonn, Germany.
E-mail: christa.mueller@uni-bonn.de, **Fax**: +49-228-73-2567

Table of contents

Scheme 1. Synthesis of SAR 03004 p.2
Fig. S1. Michaelis-Menten representation of the hydrolysis of artificial substrates (*p*-Nph-5'-TMP and *p*-Nph-5'-AMP) by human NPP1 p.4
Fig. S2. Hanes-Woolf plots of human NPP1 inhibition by non-nucleotidic and nucleotidic inhibitors p.5

Scheme 1. Synthesis of SAR 03004 (*N*-[2-[1-(6,7-dimethoxyquinazolin-4-yl)piperidin-4-yl]ethyl] sulfuric diamide) [1,2]

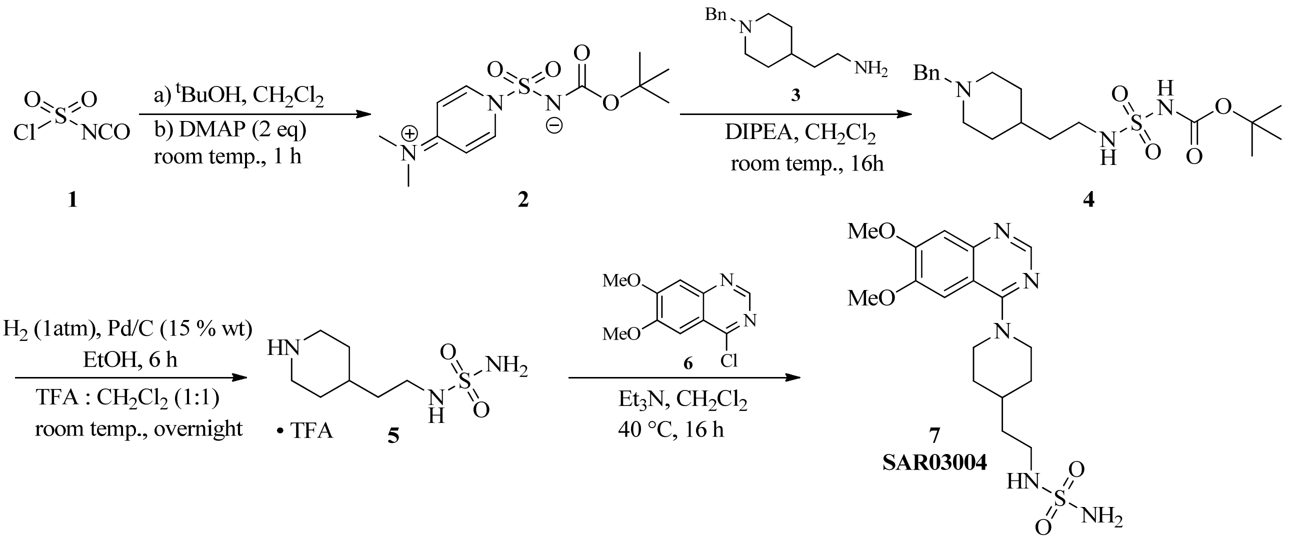

Procedure for the synthesis of (*tert*-butoxycarbonyl)-((4-(dimethylimino)pyridin-1-(4*H*)-yl)-sulfonyl)amide (**2**)

Chlorosulfonyl isocyanate (**1,** 1.2 mL, 1 eq.) was added dropwise to a cold solution of ^tBuOH (1.3 mL, 1 equiv) in anhydrous CH_2Cl_2 (15 mL). Then 4-dimethylaminopyridine (DMAP) (3.45 g, 2 equiv) was added. The mixture was stirred for 1 h at the room temperature and washed with water (3 x 15 mL). The organic layer was dried on anhydrous Na_2SO_4 and concentrated in vacuum. The colorless powder was then crystallized from CH_3CN to afford compound **2** in 80 % yield. Yield: 3.3 g (80 %), colorless solid; ^1H NMR (500 MHz, DMSO-d_6) δ (ppm) 8.45 (d, J = 8.0 Hz, 2H, $CH_{dihydropyridine}$), 6.97 (d, J = 8.0 Hz, 2H, $CH_{dihydropyridine}$), 3.22 (s, NCH_3, 6H), 1.25 (s, CH_3, 9H). ^{13}C NMR (125 MHz, DMSO-d_6) δ 156.3 (C=O), 152.2 (C_q), 141.4 ($CH_{dihydropyridine}$, 2C), 107.0 ($CH_{dihydropyridine}$, 2C), 77.9 ($C_{q,}$ CCH_3), 39.5 (NCH_3, 2C), 28.3 (CH_3, 3C); LC/ESI-MS (m/z): 302.2 $[M+H]^+$; Purity: 86.4 %

Procedure for the synthesis of *tert*-butyl-*N*-(2-(1-benzylpiperidin-4-yl)ethyl)sulfamoyl-carbamate (**4**): 2-(1-benzylpiperidin-4-yl)ethanamine (**3,** 362 mg, 1.66 mmol, 1 equiv) was dissolved in 4 mL of CH_2Cl_2 and to it 322 mg (2.49 mmol, 2 equiv) *N*,*N*-diisopropylethylamine (DIPEA) was added. One minute later, 500 mg of **2** was added and the reaction mixture was stirred at room temperature for 16 h. After completion of the reaction the solvent was removed under vacuum and the crude product was purified by flash chromatography (CH_2Cl_2 : MeOH = 9 : 1) to obtain 510 mg of pure product **4**. 510 mg (77 %), colorless solid; ^1H NMR (500 MHz, DMSO-d_6) δ (ppm) 7.36 – 7.21 (m, 5H, CH_{aromat}), 3.46 (s, CH_2Ph, 2H), 2.88 (dd, J = 13.3 Hz, J = 6.7 Hz, $CH_{2\ aliphat}$, 2H), 2.78 (d, J = 11.6 Hz, $CH_{2\ morpholine}$, 2H), 1.94 (dd, J = 11.6 Hz, J = 9.6 Hz, $CH_{2\ morpholine}$, 2H), 1.58 (d, J = 11.8 Hz, 2H), 1.40 (s, 9H, CH_3), 1.36 (J = 11.6 Hz, J = 9.6 Hz, $CH_{2\ morpholine}$, 2H), 1.34 – 1.26 (m,

$CH_{morpholine}$, 1H), 1.11 (J = 13.3 Hz, J = 6.7 Hz, $CH_{2aliphat}$, 2H); ^{13}C NMR (125 MHz, DMSO-d_6) δ 151.3 (C=O), 138.1 (C_q), 129.2 (CH_{aromat}, 2C), 128.4 (CH_{aromat}, 2C), 127.2 (CH_{aromat}), 80.9 (C_q, CCH_3), 62.5 (CH_2Ph), 53.2 ($CH_{2\ piperidine}$, 2C), 40.6 ($CH_{2\ aliphat}$), 35.4 ($CH_{piperidine}$), 32.4 ($CH_{2\ aliphat}$), 31.6 ($CH_{2piperidine}$, 2C), 28.0 (CH_3, 3C); LC/ESI-MS (m/z): 398.3 $[M+H]^+$; 396.3 $[M-H]^-$; Purity: 95.3 %

Procedure for the synthesis of *N*-[2-[1-(6,7-dimethoxyquinazolin-4-yl)piperidin-4-yl]-ethyl]sulfuric diamide) (7, SAR03004): Compound **4 (**200 mg, 0.50 mmol, 1 equiv) was dissolved in 6 mL of EtOH and to it 30 mg of Pd/C was added. The reaction mixture was allowed to stir for 6 h at room temperature under 1 atm of H_2 pressure. The reaction mixture was filtered over Celite® and the filtrate was concentrated under vacuum. The residue was dissolved in 6 mL of a 1:1 TFA : CH_2Cl_2 mixture and stirred overnight at room temperature. The crude reaction mixture was concentrated and dissolved in 6 mL of CH_2Cl_2, and to it to triethylamine (253 mg, 5 equiv) and 224 mg (1 equiv) of 4-chloro-6,7-dimethoxyquinazoline (**6**) was added. The reaction mixture was heated to reflux for 16 h. After completion of the reaction the solvent was removed and the residue was dissolved in EtOAc (10 mL) and washed with water (3 x 5 mL). The aqueous layer was extracted with EtOAc (3 x 10 mL). The combined organic layer was dried with anhydrous Na_2SO_4, filtered, and concentrated under vacuum. The crude product was purified by flash chromatography (CH_2Cl_2 : MeOH = 9.5 : 0.5) to obtain a viscous oil. This was dissolved in a small quantity of warm EtOAc, and cyclohexane was added until a faint turbidity was visible. Cooling in a refrigerator afforded the precipitation of the colorless solid product **7**. Yield: 90 mg (45 % over two steps), colorless solid; ^1H NMR (500 MHz, DMSO-d_6) δ (ppm) 8.50 (s, CH_{aromat}, 1H), 7.18 (s, CH_{aromat}, 1H), 7.10 (s, CH_{aromat}, 1H), 6.44 (s, 2H, NH_2), 6.41 (t, J = 6.0 Hz, 1H, NH), 4.13 (d, J = 13.2 Hz, $CH_{2\ piperidine}$, 2H), 3.91 (s, OCH_3, 3H), 3.89 (s, OCH_3, 3H), 3.01 (td, J = 13.2 Hz, J = 1.7 Hz, $CH_{2\ aliphat}$, 2H), 2.95 (dd, J = 13.6 Hz, J = 6.8 Hz, $CH_{2\ aliphat}$, 2H), 1.80 (d, J = 12.8 Hz, $CH_{2\ piperidine}$, 2H), 1.72 – 1.64 (m, $CH_{piperidine}$, 1H), 1.51 – 1.45 (m, $CH_{2\ piperidine}$, 2H), 1.38 – 1.33 (m, $CH_{2\ piperidine}$, 2H). ^{13}C NMR (125 MHz, DMSO-d_6) δ 161.3 (C_q), 158.3 (C_q, 2C), 155.7 (C_q), 148.4 (C_q), 118.5 (CH_{aromat}), 116.1 (CH_{aromat}), 105.6 (CH_{aromat}), 56.4 (OCH_3), 56.0 (OCH_3), 49.6 ($CH_{2\ piperidine}$, 2C), 35.2 ($CH_{2\ aliphatic}$), 32.5 ($CH_{2\ aliphatic}$), 31.8 ($CH_{2\ piperidine}$, 2C), 22.0 ($CH_{aliphatic}$); LC/ESI-MS (m/z): 396.3 $[M+H]^+$; 394.5 $[M-H]^-$; Purity: 96.7 %

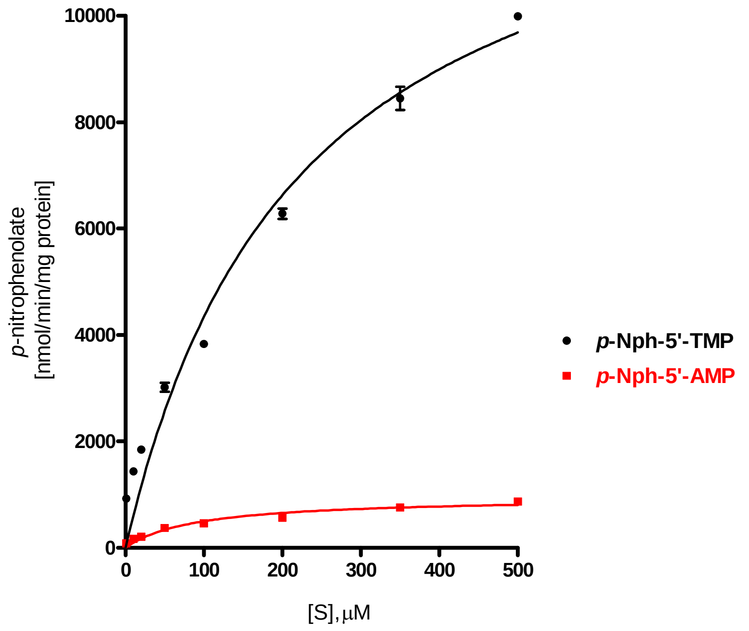

Fig. S1. Michaelis-Menten representation of the hydrolysis of artificial substrates (*p*-Nph-5'-TMP and *p*-Nph-5'-AMP) by human NPP1. Data points represent means ± SD from three separate experiments. The determined K_m values were 222 µM for *p*-Nph-5'-TMP and 188 µM for *p*-Nph-5'-AMP. The determined k_{cat} values were 22.3 s^{-1} for *p*-Nph-5'-TMP and 2.51 s^{-1} for *p*-Nph-5'-AMP.

A. ATP as a substrate

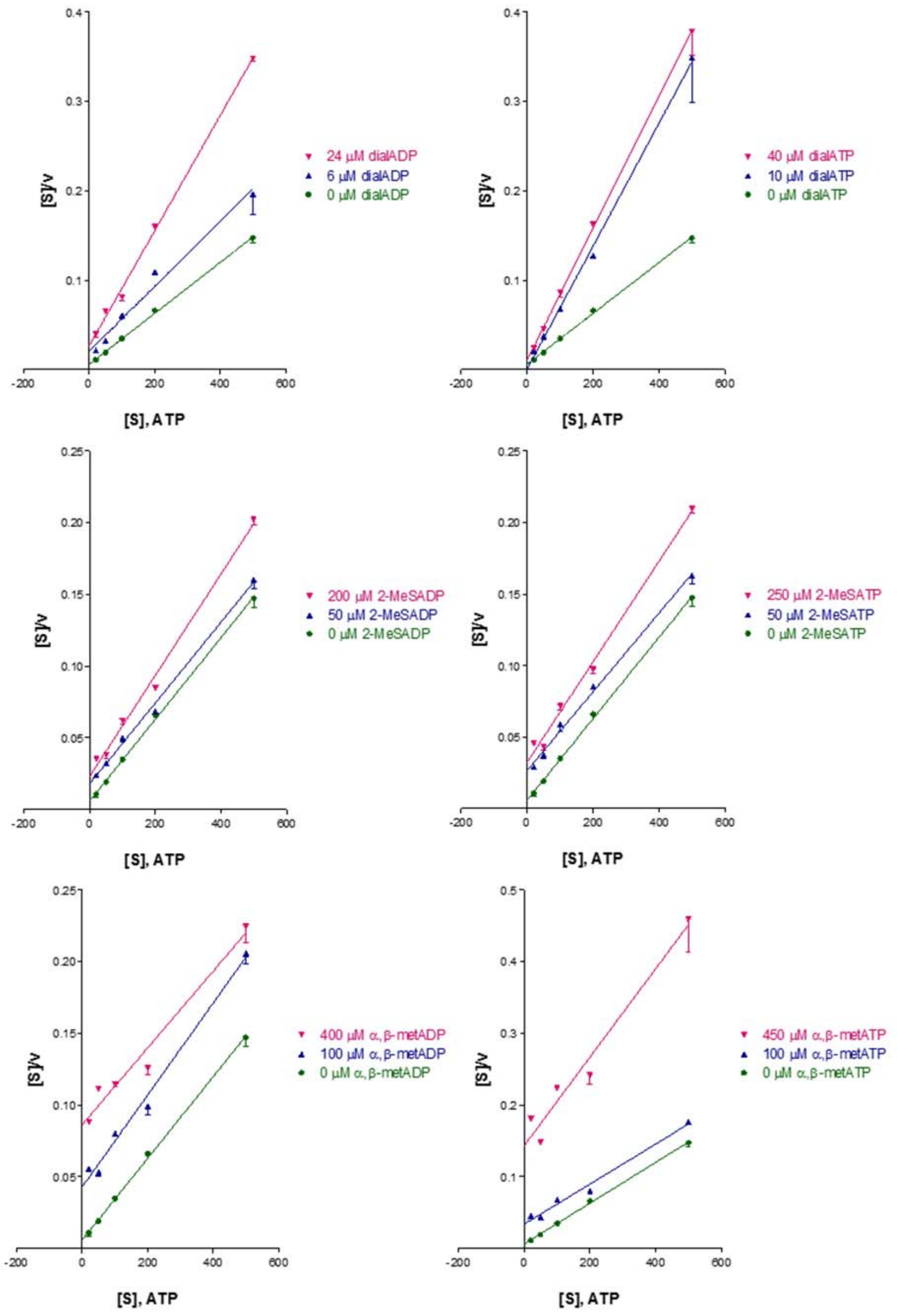

Fig. S2. Hanes-Woolf plots of human NPP1 inhibition by non-nucleotidic and nucleotidic inhibitors (**A**: ATP as a substrate, **B**: *p*-Nph-5'-TMP as a substrate, **C**: *p*-Nph-5'-AMP as a substrate). S, concentration of substrate. Data points represent means ± SD from three independent experiments.

Fig. S2 Continued

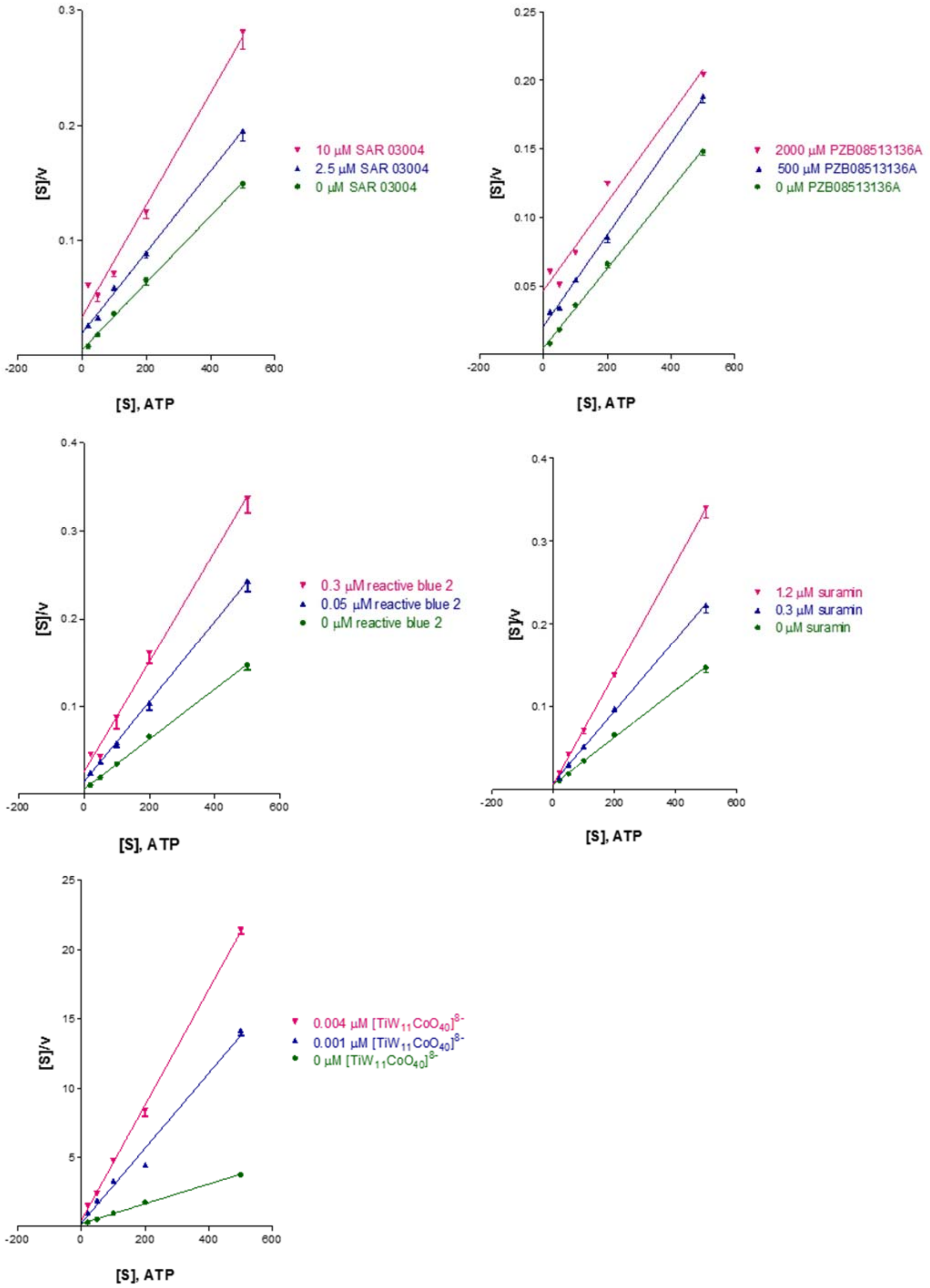

Fig. S2 Continued

B. *p*-Nph-5'-TMP as a substrate

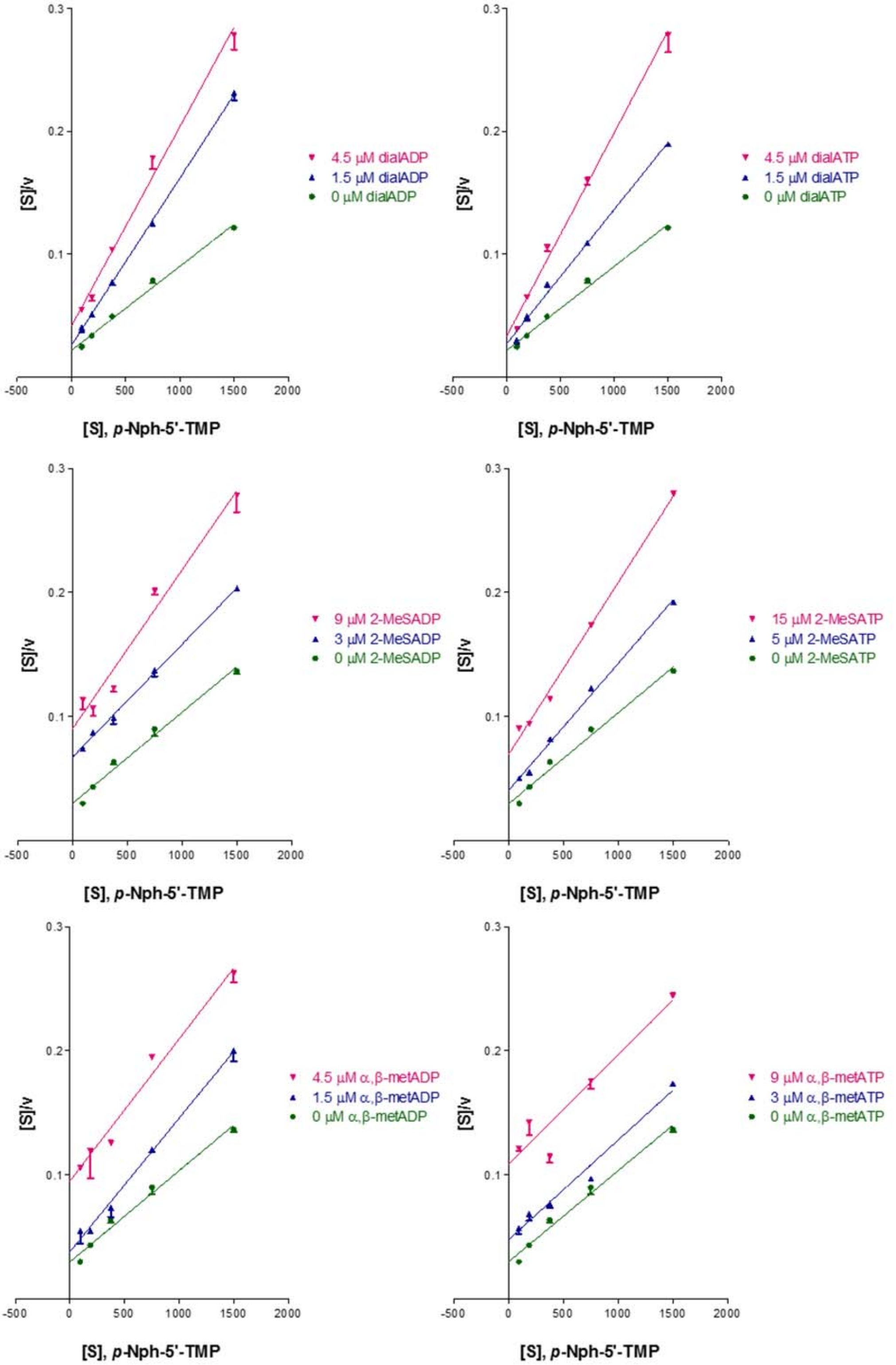

Fig. S2 Continued

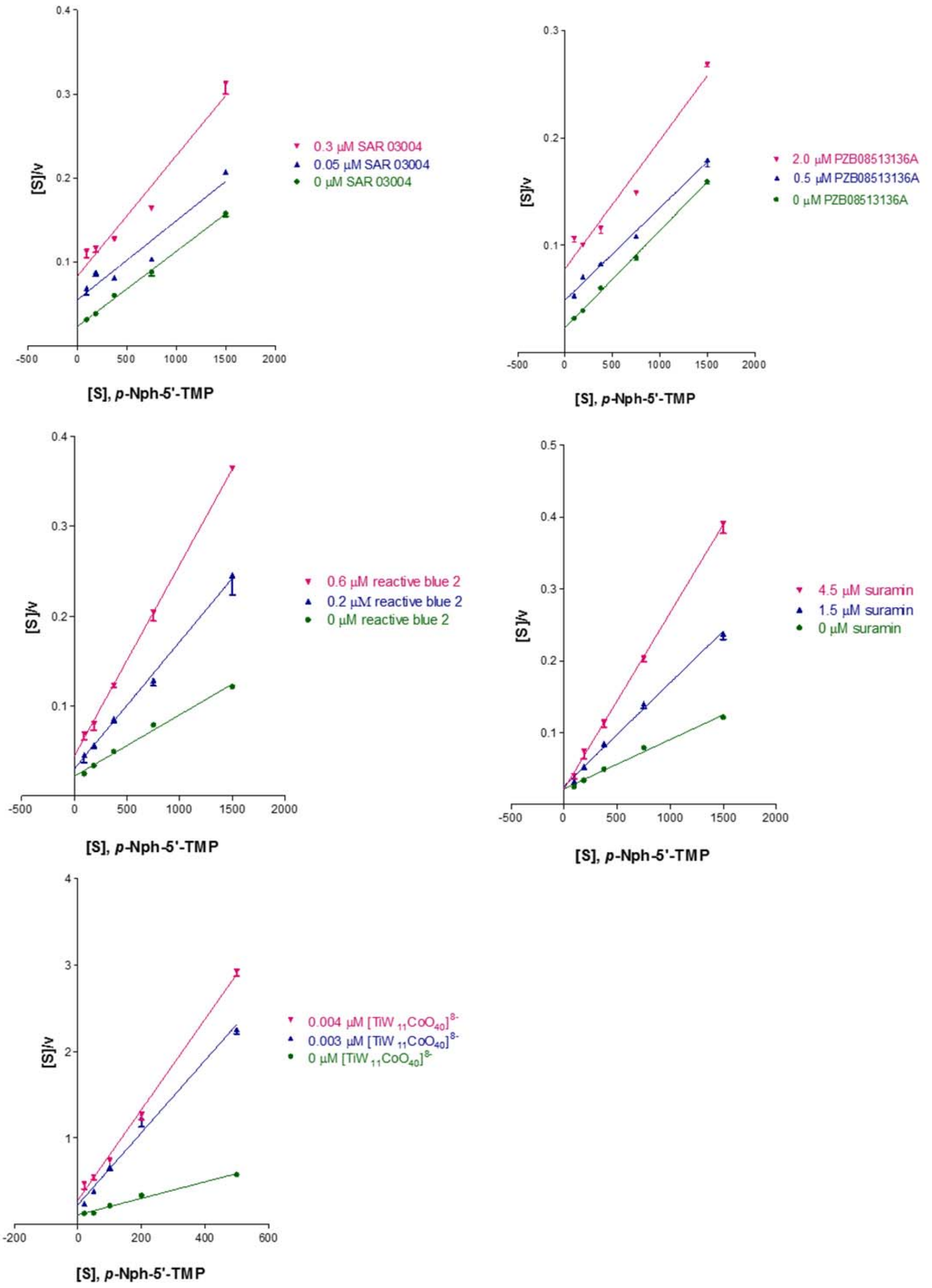

Fig. S2 Continued

C. *p*-Nph-5'-AMP as a substrate

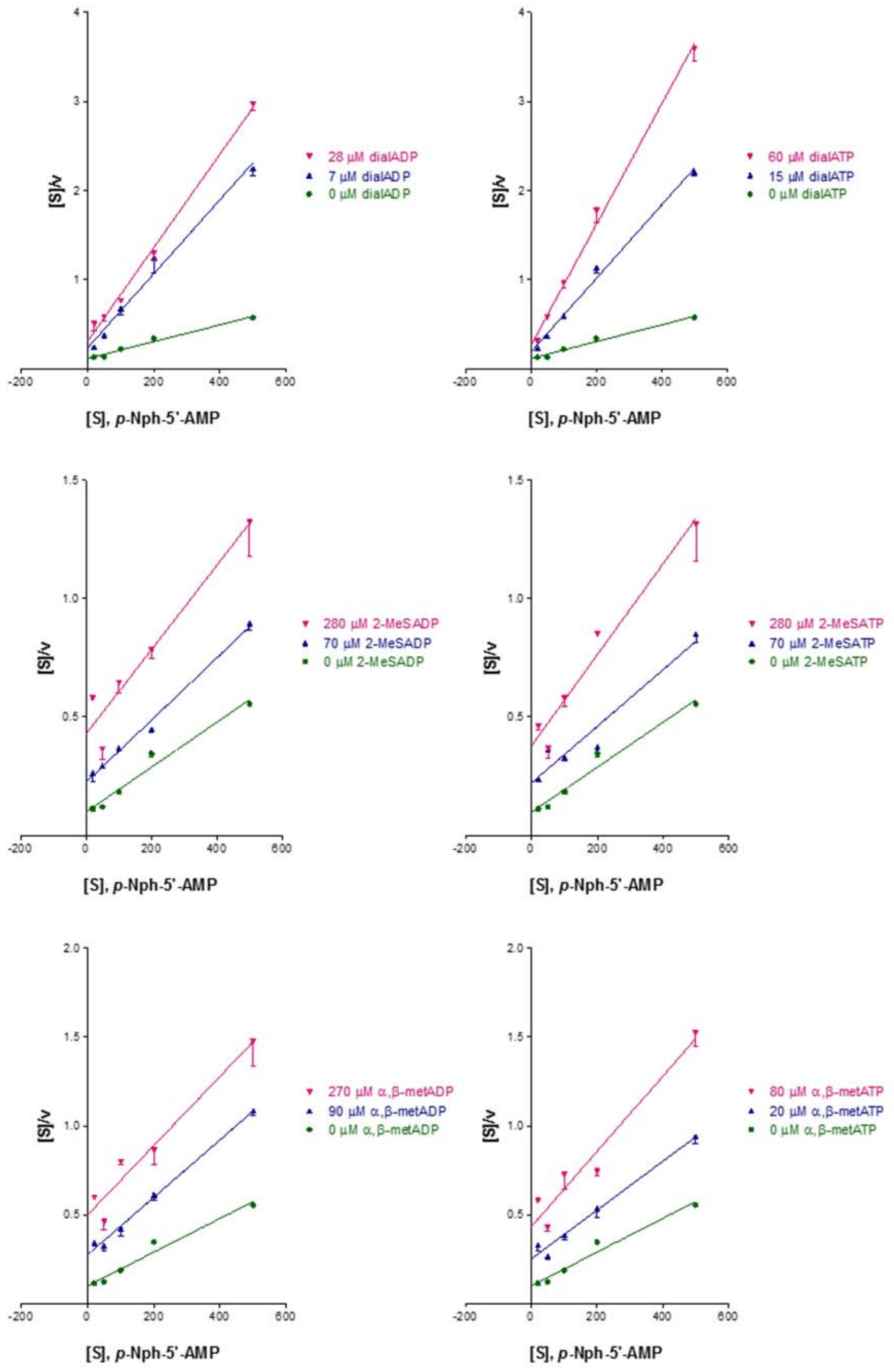

Fig. S2 Continued

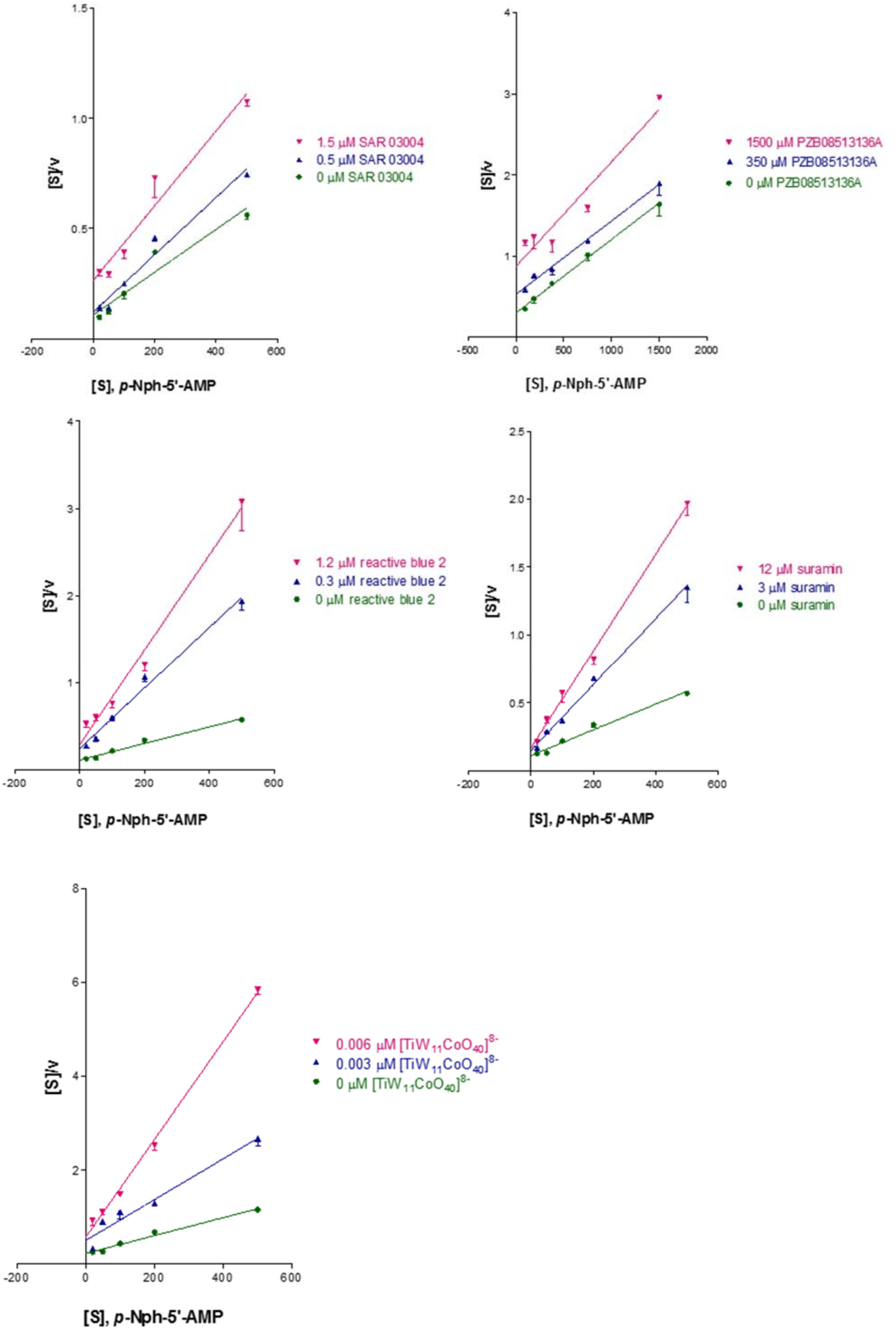

References

[1] Patel SD, Habeski WM, Cheng AC, de la Cruz E, Loh C, Kablaoui NM. Quinazolin-4-piperidin-4-methyl sulfamide PC-1 inhibitors: alleviating hERG interactions through structure based design. Bioorg Med Chem Lett 2009;19:3339–43.

[2] Winum J, Toupet L, Barragan V, Dewynter G, Montero J. N-(tert-butoxycarbonyl)-N-[4-(dimethylazaniumylidene)-1,4-dihydropyridin-1-ylsulfonyl]azanide: a new sulfamyolating agent. structure and reactivity toward amines. Org Lett 2001;3:2241–3.
